# Selective Antiproliferative Effects of Marine Oils on Neuroblastoma Cells in 3D Cultures

**DOI:** 10.3390/md23070268

**Published:** 2025-06-26

**Authors:** Luís Freiría-Martínez, Jose María Oliva-Montero, Ainhoa Rodríguez-Tébar, Ola Hermanson, Santiago P. Aubourg, Carlos Spuch, Isabel Medina

**Affiliations:** 1 Chemistry of Marine Products, Department of Food Technology, Institute of Marine Research (IIM-CSIC), C/Eduardo Cabello 6, 36208 Vigo, Spain; saubourg@iim.csic.es (S.P.A.); medina@iim.csic.es (I.M.); 2 Translational Neuroscience Group, Galicia Sur Health Research Institute (IIS Galicia Sur), SERGAS-UVIGO, CIBERSAM, Red de Investigación en Atención Primaria de Addicciones (RIAPAd), 36312 Vigo, Spain; carlos.spuch@iisgaliciasur.es; 3 Department of Biomedicine, Biotechnology and Public Health, Faculty of Medicine, Cadiz University, 11009 Cádiz, Spain; jose.oliva@uca.es; 4 Department of Neuroscience, Karolinska Institutet, Biomedicum, 17177 Stockholm, Sweden; ola.hermanson@ki.se; 5 Microscopy Service, Galicia Sur Health Research Institute (IISGS), 36216 Vigo, Spain; microscopia.iisgs@sergas.es

**Keywords:** *ω-3* PUFA, marine oils, bioactive compounds, neuroblastoma, antiproliferative treatments, sustainable development

## Abstract

Dietary marine lipids enriched in *ω-3* polyunsaturated fatty acids (PUFAs) are spotlighted for favorable effects in neurodegenerative conditions and tumor cell proliferation. Commercial marine oils, with high EPA and DHA content, consist of non-polar lipids constituted by triacylglycerols or polar oils composed of phospholipids. Both classes have shown different activities to significantly inhibit proliferation and migration, and induce apoptosis in cancer cells. This work was aimed at testing marine oils’ associated effects on neuroblastoma (NB) and glioblastoma (GB). Commercial non-polar and polar marine oils were studied in 3D spheroid models developed with human neuroblastoma, GB, and non-nervous embryonic kidney cell lines. This study also included results provided by a new sustainable polar marine oils source: fishery side-streams. Cell viability and mitochondrial activity assessments demonstrated that both marine oils dramatically reduced NB cells’ metabolism, proliferation, and viability. Effects on GB and epithelial cells were different, including a metabolic increase. Marine oils also induce cell differentiation and selectively modulate the activity of neurons and glia, depending on the oils’ chemical form. Sustainable polar oil showed bioactive characteristics similar to commercial krill oil. We propose that marine oils rich in triacylglycerols and phospholipids with high EPA and DHA levels may be a useful tool in NB antiproliferative therapies.

## 1. Introduction

**Figure 1:**
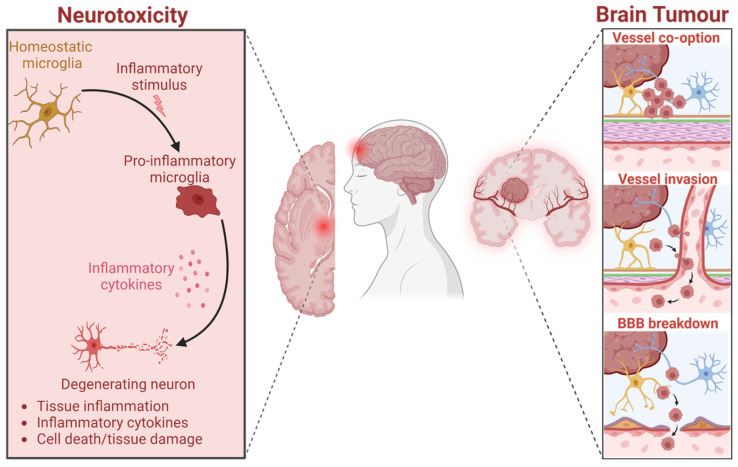
Neuroinflammation is a common hallmark for neurodegenerative pathologies and tumoral. Created in BioRender.com.

Regular consumption of *ω-3* polyunsaturated fatty acids (PUFAs), mainly eicosapentaenoic acid (EPA) and docosahexaenoic acid (DHA), has been associated with the inhibition of cancer development and malignant cell growth in vitro and in vivo [1]. In this beneficial effect, the role of *ω-3* PUFA on the resolution of inflammation seems to be decisive [2]. Inflammation has a physiological role, and under normal circumstances, it is self-limiting in regard to the induction of active resolution processes. However, failure to resolve inflammation can result in excessive, inappropriate, or ongoing processes that can cause irreparable damage to host tissues, leading to pathology and disease [3]. Non-resolving inflammation is a component of the tumor microenvironment, being in part a cause and not only an effect of cancer development [4,5,6] (Figure 1). The cancer cell embodies characteristics that permit survival beyond its normal life span and allow it to proliferate abnormally. Lack of normal growth control is not only operative in early tumorigenesis but also during tumor metastasis [7].

Brain tumor development induces the modulation of peripheral cerebral tissue, suppressing the immune response and leading to inflammatory events in the tumoral microenvironment [8]. This unusual inflammation is largely associated with the malignant transformation of low-grade gliomas and other brain tumors, inducing activation of transcription factors, which contribute to cancer cell propagation [9]. Immunosuppression and inflammatory changes can induce tumoral cell invasion and metastasis. Radiotherapy in patients with brain cancer or metastases can also contribute to neuroinflammation and necrotic lesions.

Glioblastoma (GB) is the most aggressive primary malignant brain cancer in adults [8]. Neuroinflammatory damages induce a series of events, such as a decrease in neuronal signaling, plasticity, and axonal regression, triggering demyelination, and originate myelin fragments that can activate microglia and induce inflammatory responses [10]. But microglia themselves, as innate immune cells of the central nervous system (CNS), play key roles in mediating these neuroinflammatory responses. In many circumstances, including CNS injury, some neuroinflammatory responses are balanced with intrinsic repair processes that influence functional recovery [11]. Research in cellular and animal models has pointed out the preventive effect of DHA on neuroinflammation by modulating glial cell activity [12]. DHA has largely been demonstrated to attenuate inflammatory responses through inhibition of microglia-mediated neuroinflammation in the CNS by decreasing pro-inflammatory cytokines and chemokines [13].

Neural tissue contains the highest concentrations of the most highly unsaturated PUFAs found in mammals, with DHA and arachidonic acid (AA) as the two major PUFAs [14]. DHA is the most predominant fatty acid (FA) found at the sn-2 position in brain phospholipids (PLs) on neuronal and synaptic membranes [15]. It is largely required for normal neural development and function [16]. Previous research suggests that neural cancer cells such as neuroblastoma (NB), glioma, and meningioma show a significant lack of DHA content compared to normal nervous tissue, whereas the level of AA reveals a notable increase [17].

In addition to their essential role in forming part of the brain cell membranes, the imbalance between *ω-3* and *ω-6* PUFA in nervous system tumors may largely affect the formation of lipid mediators involved in the resolution of inflammation [3,18]. These resolvers are part of the huge family of lipid metabolites formed by the metabolism of PUFA by the action of lipoxygenase, cyclooxygenase, and cytochrome P450 monooxygenase enzymes [19]. In general, AA-derived lipid mediators are proinflammatory, e.g., prostaglandins and leukotrienes of the 2- and 4-series, whereas products of *ω-3* PUFA are resolvers of inflammation (resolvins, maresins, and protectins) [18]. Several of the *ω-6*-derived prostaglandins and leukotrienes have been shown to promote tumor growth, whereas inhibition of enzymes that are responsible for their production suppresses NB growth. DHA induces apoptosis of NB cells by mechanisms involving intracellular accumulation of cytotoxic DHA-derived metabolites [20].

Several studies in different types of brain cancer, primarily glioma, have shown that the presence of *ω-3* PUFA lipid mediators downregulates tumor growth, proliferation, angiogenesis, and metastasis [21,22]. Meanwhile, *ω-6* PUFA lipid mediators induce an increase in these pro-tumor processes [23]. According to the resolving effects induced by *ω-3* PUFA, supplementation with marine oils has also shown antiproliferative effects on different cancer cell lines [24,25]. Other *ω-3* PUFAs, such as γ-linolenic acid (GLA), have decreased proliferation and induced apoptosis in spheroids from several glioma cell lines: C6, U87, U373, and MOG-G-CCM [26,27]. Outside of their role in the CNS, DHA supplementation also attenuated the hyperproliferation of keratinocytes in a 3D tissue-engineered psoriatic skin model [28], and *ω-3* FA blocked prostate tumor spheroid growth [29].

In the form of EPA and DHA, *ω-3* PUFA can be directly submitted through the diet, mainly resulting from marine oils, fish, algae, and seafood consumption. After ingestion, DHA supplied mainly by the liver is incorporated into the brain from the blood. After being released from serum albumin, it can cross the blood–brain barrier (BBB) and be incorporated into the cells [30]. However, incorporation seems to be regulated, and animal research in cultured neural cells supplemented with DHA suggested that DHA entrance and synthesis in the brain might be sensed and regulated, as previously shown in retinal pigment epithelium [31]. Nevertheless, questions related to the effective dose and the best window of time for application, and, definitively, knowledge of the mechanisms of action underlying the effect of *ω-3* PUFA in brain cancer or brain metastases are still unclear.

An important feature of *ω-3* PUFA consumption is related to the chemical form in which EPA and DHA are being ingested. Consumption of *ω-3* PUFA occurs mainly through the intake of fish or fish oil supplements, where the *ω-3* PUFA forms are esterified to glycerol of triacylglycerols (TGs) or PLs, and the content and proportion of them are different depending on the marine source [32,33]. PLs are reported to concentrate a high *ω-3* PUFA proportion, and they are more stable than TGs. Also, PL-bound FAs are more efficiently absorbed and incorporated into cell membranes. Benefits might be due to the structural differences between the two oils, exerting their functional properties through specific mechanisms of action [33]. Among marketed marine oils, non-polar oils formed mainly by TGs are widely spread, but commercial sources for polar marine oils with a high percentage of PLs are less common. The recovery of marketable by-products from fish wastes is currently an important food waste-reduction strategy [34]. For many marine species, the regular cleaning, dressing, and processing produce high quantities of by-products that are rich in many nutrients [35]. Notably, the highest contents of high-added-value compounds are often included in body parts of marine organisms that are commonly discarded [36,37]. Among marine species, cephalopod catches have gradually increased in the last decade due to a growing market demand and an expansion of fisheries into new fishing grounds and deeper waters [38]. Therefore, great attention has been accorded recently to the obtention of bioactive compounds like phospholipids, *ω-3* PUFA, and tocopherols from cephalopod by-products [39,40].

In conventional tissue culture, cells are grown in a 2D environment, which can alter their gene expression, signaling, and morphology compared to in vivo conditions, potentially compromising clinical relevance [41]. Transitioning from 2D to 3D cell cultures is driven by the need for models that more accurately mimic the functions of living tissues [42]. In a 3D tissue, concentration gradients can be formed for various soluble components in the culture medium, ranging from basic nutrients to effector molecules [43]. Three-dimensional cell cultures reestablish physiological cell–cell and cell–ECM interactions, better replicating real tissue specificity compared to traditional 2D cultures [42]. It has been suggested that the future of cell culturing for fundamental studies and biomedical applications thus lies in the creation of multicellular structure and organization in 3D [44].

The human brain contains a wide variety of specialized cells, each with specific functions. These cells can be divided into two main categories: neurons and glial cells. Neurons can be of many different types, forming highly complex networks that enable them to perform their functions efficiently. Glial cells outnumber neurons and play important roles in support, protection, maintenance, synapse formation, and even the transmission of information itself.

This work was aimed at investigating the effects of marine oils enriched with *ω-3* PUFA using a 3D cell culture model (“spheroids”) of neuronal and glial tumoral cells. Since the chemical form of the fatty acids in the glycerol backbone seems to be determinant, the study involves marine oils coming from different natural sources and varying the chemical form of the esterified fatty acids. Then, the biological effects of the newly sustainable produced polar and non-polar oils (from squid and fish discards and having a high content of *ω-3* PUFA esterified to PL or TG, respectively) were compared. Two commercial oils, a saturated coconut oil and a marine oil enriched in *ω-3* PUFA-PL, were used as references. A non-neural cell line, HEK293T, derived from human embryonic kidney, was used as a control.

## 2. Results and Discussion

### 2.1. Polar and Non-Polar Marine Oils

Four oils with different TG and PL amounts were used, two of them with a high concentration of TG over PL. Thus, coconut oil, with more than 99% of TG enriched in SFA, was obtained from cold-pressing unrefined organic coconut oil. Furthermore, fish oil containing 95% TG (17% EPA; 10% DHA) was obtained by blending adequate proportions of the commercially available fish oils from AFAMPES 121 EPA (AFAMSA, Vigo, Spain), EnerZona Omega 3 RX (ENERZONA, Milano, Italy), and Oligen liquid DHA 80% (IFIGEN-EQUIP 98, Barcelona, Spain). Finally, two oils with a high concentration of PL were also used. One of them, krill oil, was obtained by supercritical extraction of Antarctic krill (Euphausia superba) with 32% PL (16% EPA, 9% DHA) and provided by Rimfrost AS (Ålesund, Norway). The second one, squid oil, 43% PL (16% EPA, 30% DHA), was extracted with ethanol from squid glands, under the conditions described by Aubourg et al. [45]. Table 1 shows the FA composition of all four oils. Table 2 shows the % of lipid classes for all four oils.

The potential action of *ω-3* PUFA-enriched oils on a neuron cell type SH-SY5Y, obtained from NB, and a glial-type cell line, U251, coming from GB was firstly checked through cell viability and IF assays. A non-neural cell line, HEK293T, derived from human embryonic kidney, was used to confirm the absence of negative effects on normal or non-cancer cells.

### 2.2. Effect of Polar Marine Oils on NB Cell Viability

**Figure 2:**
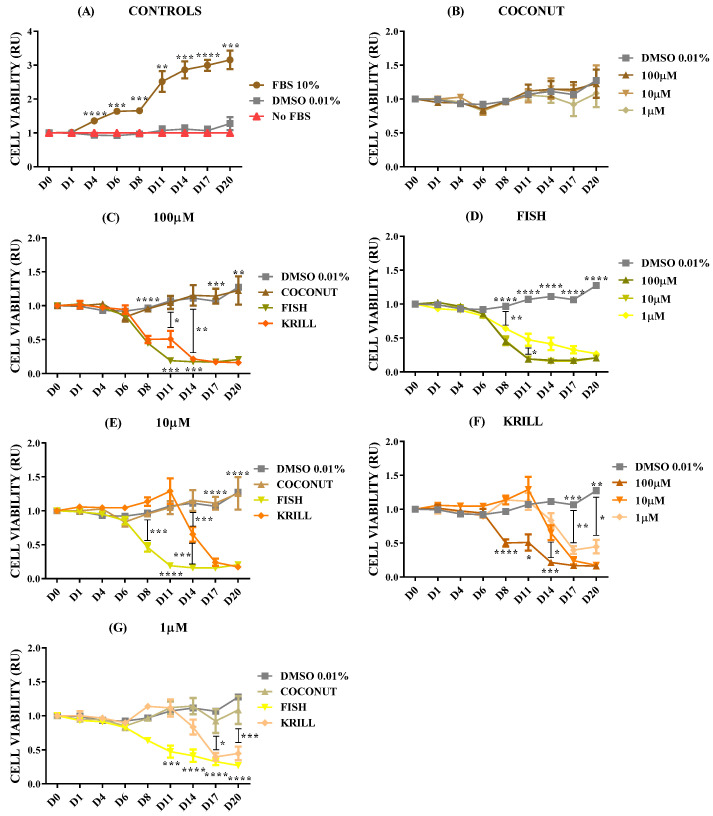
Cell viability relative units (RU) in SH-SY5Y spheroids according to oil types and concentrations. n = 8. (**A**) Control spheroids in FBS 10%, DMSO 0.01%, and no FBS. (**B**) Spheroids in DMSO 0.01% and coconut oil. (**C**) Spheroids in DMSO 0.01% and 100 µM coconut, fish, and krill oils. (**D**) Spheroids in DMSO 0.01% and fish oil. (**E**) Spheroids in DMSO 0.01% and 10 µM coconut, fish, and krill oils. (**F**) Spheroids in DMSO 0.01% and krill oil. (**G**) Spheroids in DMSO 0.01% and 1 µM coconut, fish, and krill oils. Only multiple comparisons tests are represented. ANOVA and paired *t*-test are listed in Table S1. (****) *p* ≤ 0.0001; (***) *p* ≤ 0.001; (**) *p* ≤ 0.01; (*) *p* ≤ 0.05.

The effect of marine oils was tested on NB, a neoplasm of the sympathetic nervous system which is the second most common extracranial malignant tumor of childhood and the most common solid tumor of infancy [46]. NB is a heterogeneous malignancy with remarkable heterogeneity observed in tumor phenotype, ranging from spontaneous regression to relentless progression, and with prognosis extending from near-uniform survival to high risk for fatal demise [46,47]. Figure 2 shows the dose-dependent effect for cell viability in SH-SY5Y spheroids in coconut oil, fish oil, and krill oil at three different concentrations. Before determining cell viability, 3D spheroids were left for at least 3–4 days in complete culture media (FBS 10%) to let individual cells proliferate and form aggregates. It also assesses if cells maintain their viability after gel formation.

The results for control SH-SY5Y spheroids (Figure 2A) showed a clear and significant increase in cell metabolism when cultured in optimal conditions (FBS 10%) in comparison with negative culture controls cultivated without FBS or with DMSO 0.01% (dissolving agent for oils). The addition of the saturated fatty acid oil, coconut oil, did not provoke any effect on cell viability of NB at any of the concentrations tested (Figure 2B). However, supplementation of both marine oils enriched in *ω-3* PUFA produced clear changes in NB spheroids’ viability. Fish oil with a major content of *ω-3* PUFA in the form of TG caused a significant and almost complete reduction of cell metabolism from day 6 on, and this effect was not dependent on oil concentration (paired *t*-test Fish 10 µM - 1 µM, *p* = 0.0710; Figure 2D). Supplementation of krill oil, with a major content of *ω-3* PUFA in the form of PL, provoked a dose-time-dependent effect, showing a complete reduction of cell metabolism from the sixth day, when applied at 100 µM. When krill oil was used at 10 µM and 1 µM, the results revealed a reduction of cell viability, but with less efficiency than 100 µM (Figure 2F).

According to the above results, there were significant differences in the outcomes provoked by the addition of the three oils. Coconut oil with no PUFA did not show any effect on cell viability at any tested concentration (Figure 2B). However, both marine oils, krill oil and fish oil, were effective in inhibiting cell viability at any concentration. Fish oil demonstrated an earlier effectiveness for decreasing cell viability than krill oil at 1 (Figure 2G) and 10 µM (Figure 2E). The most concentrated dose, 100 µM (Figure 2C), addressed similar inhibitory effects by both marine oils.

**Figure 3:**
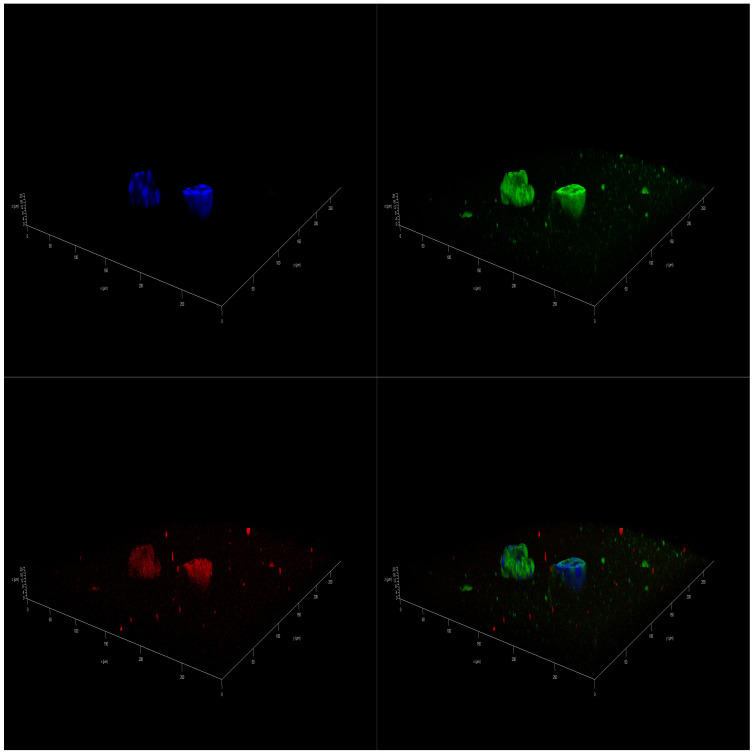
IF 3D images of SH-SY5Y spheroids on day 14 (D14) of being in culture medium with 100 µM of fish oil. Spheres are stained with DAPI^TM^ for the nucleus in blue, Alexa 488 for β3-tubulin in green, and MitoTracker^TM^ in red.

**Figure 4:**
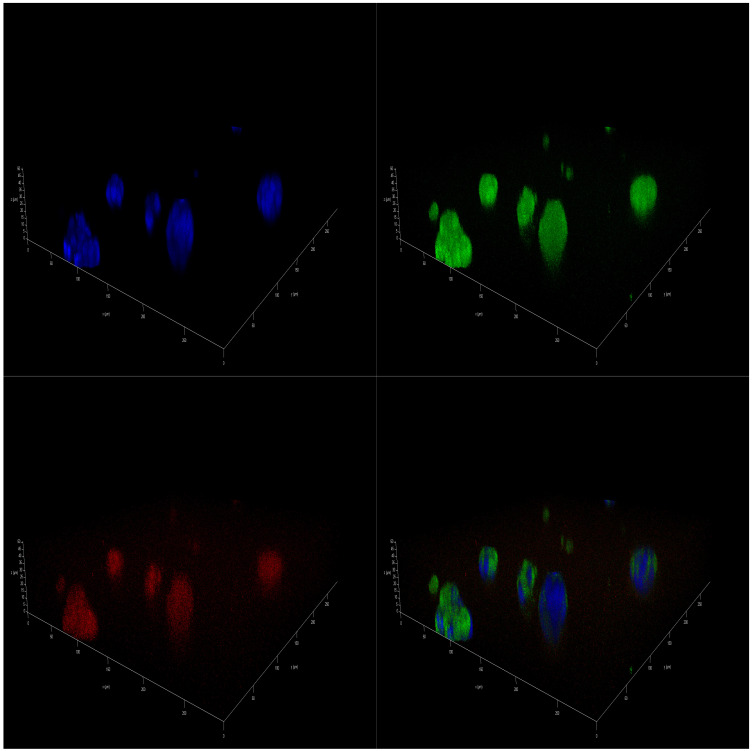
IF 3D images of SH-SY5Y spheroids on day 14 (D14) of being in culture medium with 100 µM of krill oil. Spheres are stained with DAPI^TM^ for the nucleus in blue, Alexa 488 for β3-tubulin in green, and MitoTracker^TM^ in red.

Immunofluorescence (IF)-staining images corroborated the presence of a differential effect of marine oils on NB. Confocal laser scanning microscopy analysis of mitochondria of SH-SY5Y cells that were stained revealed a higher intensity in accordance with a higher value of the mitochondrial activity. In addition, there was a significant increase in the size of the spheres cultivated in FBS 10% from the initial day, D0 sample (Figure S1), to day 14, D14 sample (Figure S2). However, spheres cultivated on DMSO 0.01% and also in coconut oil showed a decreased proliferation, leading to a final smaller size after 14 days of treatment (Figures S3 and S4). As for marine oils, fish oil and krill oil treatments reduced the size of the spheroids, and the intensity of mitochondrial staining became more diffuse (Figure 3 and Figure 4). These results indicate that those reductions in the metabolic activity and, thus, in cell viability, may be caused by mitochondrial dysfunction due to metabolism changes induced by *ω-3* dietary PUFA. In this regard, EPA and DHA treatments reduced the mitochondrial membrane potential of NB cells [48], and tumors treated with fish oil have previously displayed increased mitochondrial damage, as evidenced by the mitochondrial appearance on electron microscopy and the OxPhos complex IV subunit staining [49].

Krill oil showed less effectiveness than fish oil in reducing cell viability according to its lower effectiveness when oil was supplemented to spheroids in concentrations of 1 or 10 µM, (Figure 2E–G). Thus, the chemical form of the PUFA-enriched lipids seems to have an impact over marine oil efficacy in terms of TG acting significantly faster than PL, at lower concentrations. However, both oils lead to a similar reduction effect at a concentration of 100 µM. To check this effect, a set of experiments aimed at testing the properties of a new polar oil extracted from cephalopod biomass was executed.

**Figure 5:**
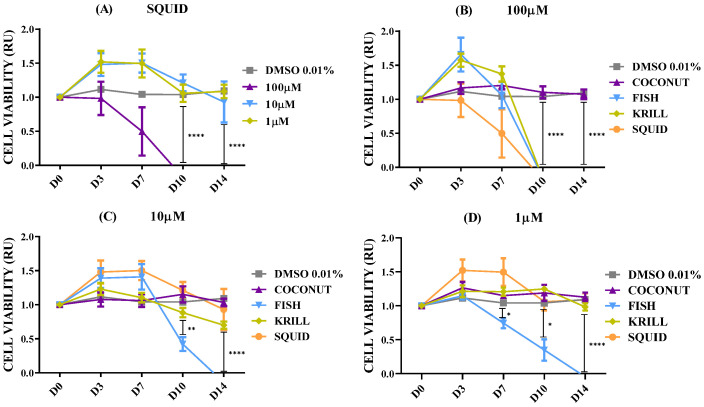
Cell viability relative units (RU) in SH-SY5Y spheroids according to squid oil and concentrations. n = 8. (**A**) Spheroids in DMSO 0.01% and squid oil. (**B**) Spheroids in DMSO 0.01% and 100 µM coconut, fish, krill, and squid oils. (**C**) Spheroids in DMSO 0.01% and 10 µM coconut, fish, krill, and squid oils. (**D**) Spheroids in DMSO 0.01% and 1 µM coconut, fish, krill, and squid oils. Only multiple comparisons tests are represented. ANOVA and paired *t*-test are listed in Table S2. (****) *p* ≤ 0.0001; (**) *p* ≤ 0.01; (*) *p* ≤ 0.05.

**Figure 6:**
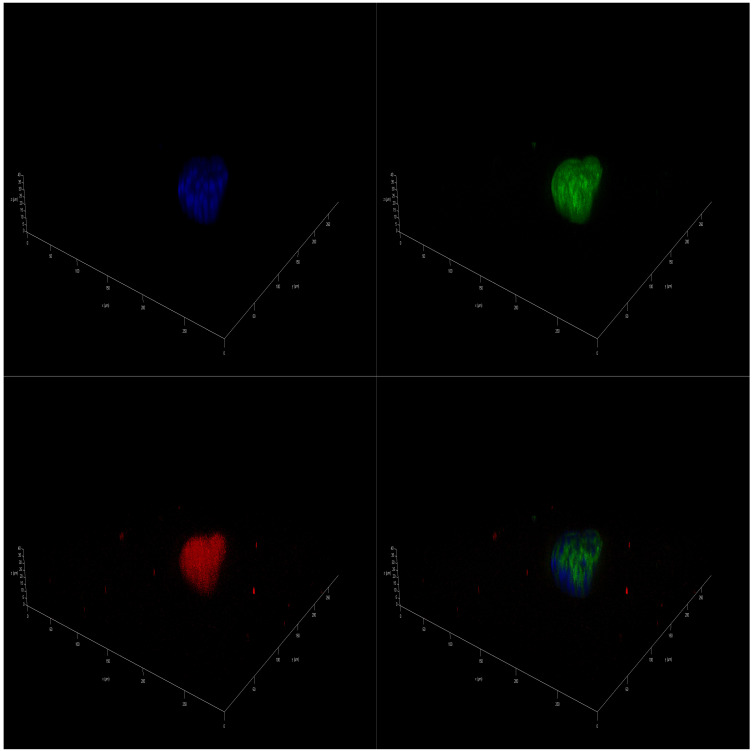
IF 3D images of SH-SY5Y spheroids on day 14 (D14) of being in culture medium with 100 µM of squid oil. Spheres are stained with DAPI^TM^ for the nucleus in blue, Alexa 488 for β3-tubulin in green, and MitoTracker^TM^ in red.

Marine oils extracted from cephalopod by-products have resulted in a convenient biomass for preparing *ω-3* PUFA concentrates [50]. The digestive gland of these species accumulates lipids, along with a notable proportion of PL in the ovary/testis [51,52], making makes these side-streams an interesting source of PL to get polar marine oils. This new oil had a high content of PL, 43%, and *ω-3* PUFA, namely EPA (16%) and DHA (30%), similar to the commercial krill oil. Figure 5A shows the detailed results corresponding to the polar oil extracted from cephalopod biomass. Data confirmed the previous findings. Coconut oil had no effect on cell viability at any concentration tested, and PUFA-enriched oils (fish, krill, and squid oils) were able to induce a reduction in cell viability. Fish oil enriched in non-polar lipids in the form of TG was more effective at lower concentrations than both polar marine oils enriched in PL. Fish oil generated a significant reduction in NB spheroids from D7 on, causing a complete collapse of neural metabolism at D14 (Figure 5B–D). Similar results were seen after treatment with krill oil and squid oil, but only for 100 µM concentration (Figure 5B). Polar marine oils supplemented at concentrations ranging between 10 and 1 µM were less effective than fish oils (Figure 5C,D). Data of IF staining images corresponded to cell lines treated with squid oil agreed with cell viability data. Figure 6 shows the spots corresponding to stained mitochondria of SH-SY5Y cells supplemented with squid oil. This revealed a reduced size and lower intensity than DMSO control (Figure S3).

**Figure 7:**
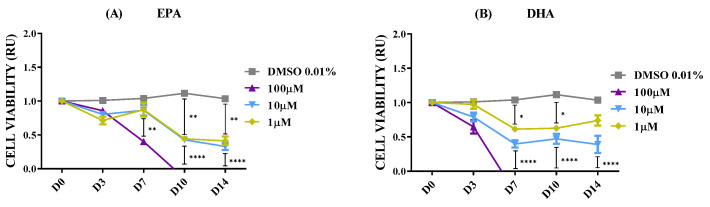
Cell viability relative units (RU) in SH-SY5Y spheroids according to isolated EPA and DHA fatty acids. n = 8. (**A**) Spheroids in DMSO 0.01% and EPA fatty acid. (**B**) Spheroids in DMSO 0.01% and DHA fatty acid. Only multiple comparisons tests are represented. ANOVA and paired *t*-test are listed in Table S3. (****) *p* ≤ 0.0001; (**) *p* ≤ 0.01; (*) *p* ≤ 0.05.

Individual EPA (Figure 7A) and DHA (Figure 7B) replicated the antiproliferative effect observed in all three oil mixtures, especially when applied at 100 µM. IF-staining images were consistent with these observations (Figures S5 and S6). At 10 and 1 µM, EPA and DHA also showed a remarkable cell viability reduction, tending toward 50%.

Results suggesting that the antiproliferative effect of *ω-3* PUFAs are consistent with previous publications that support their effectiveness in suppressing the growth of NB. The *ω-3* fatty acids DHA and EPA inhibited the proliferation of tumoral neuron cell lines in a time- and concentration-dependent manner [48,53,54]. DHA and EPA treatments downregulated the expression of CDK2 and cyclin E, suggesting that PUFAs trigger cell cycle arrest at G0/G1 phase, also decreasing the percentage of cells in the S phase [48]. Furthermore, EPA and DHA could induce NB cell death by inducing several hallmarks of apoptosis: PUFA treatment induces DNA fragmentation and phosphatidylserine externalization, increases the expression of pro-apoptotic Bax protein, and decreases the levels of anti-apoptotic Bcl-XL [48]. In addition, prolonged time of exposure to PUFA prevented the clonogenic survival of NB cells [54]. Remarkably, *ω-3* PUFA had no direct cytotoxic effect on normal neuronal cells and non-tumorigenic human cell lines such as human embryonic kidney HEK-293 cells [48].

Lipid accumulation in neurons is toxic not only because of the susceptibility of these lipids to peroxidation, but also overabundance of fatty acids may enter nonoxidative metabolic pathways, triggering excessive ceramide production that is toxic to cells [55]. Fatty acids can be converted into acylcarnitines, causing mitochondrial fragmentation and dysfunction, and ROS production [56].

### 2.3. Effect of Polar Marine Oils on GB Cell Viability

**Figure 8:**
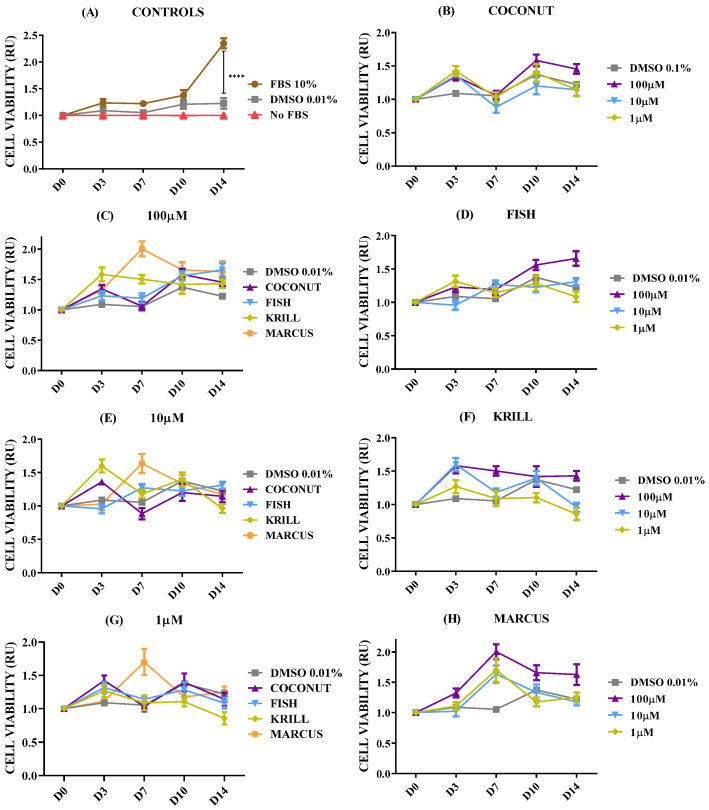
Cell viability relative units (RU) in U251 spheroids according to oil types and concentrations. n = 8. (**A**) Control spheroids in FBS 10%, DMSO 0.01%, and no FBS. (**B**) Spheroids in DMSO 0.01% and coconut oil. (**C**) Spheroids in DMSO 0.01% and 100 µM coconut, fish, krill, and squid oils. (**D**) Spheroids in DMSO 0.01% and fish oil. (**E**) Spheroids in DMSO 0.01% and 10 µM coconut, fish, krill, and squid oils. (**F**) Spheroids in DMSO 0.01% and krill oil. (**G**) Spheroids in DMSO 0.01% and 1 µM coconut, fish, krill, and squid oils. (**H**) Spheroids in DMSO 0.01% and squid oil. Only multiple comparisons tests are represented. ANOVA and paired *t*-test are listed in Table S6. (****) *p* ≤ 0.0001.

The effect of oils was then tested in the GB U251 cell line, the most common primary brain cancer in adults, mainly arising from oligodendrocyte progenitor-like cells, and it is usually fast-growing and often treatment-resistant [57,58]. Figure 8 shows the cell viability of U251 cell spheroids supplemented with the coconut, fish, krill, and squid oils after 14 days of culture. GB spheroids responded very positively to culture in complete medium (10% FBS), significantly increasing cell viability (Figure 8A). DMSO addition did not provoke any reduction in U251 glia cell viability (Figure 8B). As can be observed in Figure 8C–H, supplementation with the different fish, krill, or squid oils did not provoke any effect on cell viability, at any of the concentrations tested. None of the concentrations tested ranging from 1 µM to 100 µM showed any significant reduction in glial cell proliferation. Conversely, higher doses of PUFA *ω-3*-enriched marine oils did generate an increase in cellular metabolism of U251 spheroids that is significantly greater than DMSO controls and saturated fatty acid (SFA)-treated cells. The chemical form of the PUFA-enriched lipids, TG or PL, did not generate significant differences over cell viability. Individually added EPA or DHA replicated the effect that the rest of PUFA-enriched oils previously had (Figure S7 Table S5). These results could be a hallmark of the aggressiveness of this tumoral cell line.

**Figure 9:**
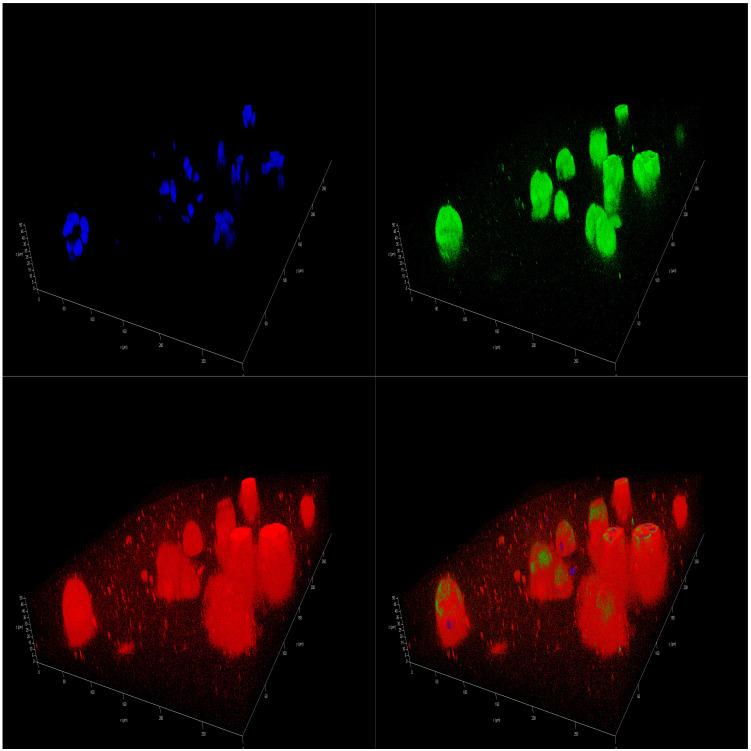
IF 3D images of U251 spheroids on day 14 (D14) in culture medium with 100 µM of fish oil. Spheres are stained with DAPI^TM^ for the nucleus in blue, Alexa 488 for GFAP in green, and MitoTracker^TM^ in red.

**Figure 10:**
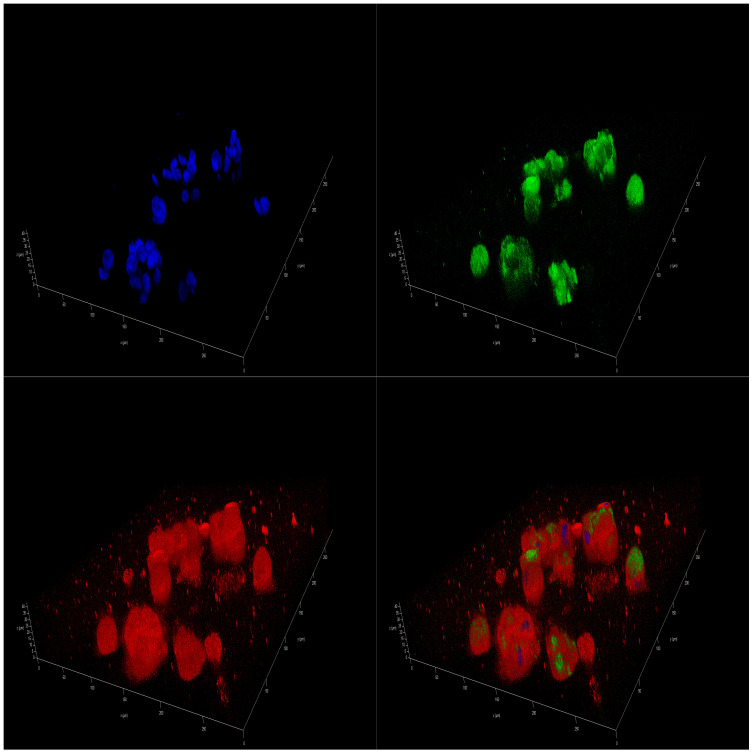
IF 3D images of U251 spheroids on day 14 (D14) of being in culture medium with 100 µM of krill oil. Spheres are stained with DAPI^TM^ for the nucleus in blue, Alexa 488 for GFAP in green, and MitoTracker^TM^ in red.

As regard to IF tests, Mitotracker^TM^-stained images revealed a higher intensity in U251 cells spheroids cultivated in complete medium (10% FBS) after 14 days (Figures S8 and S9). IF-staining spheroids corroborated the increase in mitochondrial activity in spheroids cultivated with SFA-enriched coconut oil and with the three PUFA-enriched marine oils (Figure 9, Figure 10, Figures S10 and S11).

These results, taken together, indicate that there is no antiproliferative activity of marine oils, either as TG or PL, in GB spheroids. It has previously been reported that EPA and DHA in the form of free fatty acids had a significant dose-dependent cytotoxic action on U87MG, U251MG, D54MG, and GL261 GB cells cultured in 2D. DHA raised the proportion of cells arrested in the sub-G1 phase, induced apoptosis markers such as cleavage of poly(ADP-ribose) polymerase (PARP) and DNA fragmentation, and significantly increased autophagic activity [59,60]. Additionally, increasing DHA content in the GB microenvironment may reduce the migration and infiltration of neural stem-like cancer cells [61]. For GB, there is also a bibliography highlighting the effect of *ω-6* PUFA, especially γ-linolenic acid [GLA, 18:3(*ω-6*)] [26,27,59,62]. In many tumor cells, the *ω-6* essential fatty acid metabolism is abnormal because there is a decrease in the activity of the enzyme Δ6 desaturase, which is an essential step for the formation of GLA [62]. Results indicated that the *ω-3*, *ω-6*, and *ω-9* combinations significantly reduced succinate dehydrogenase (SDH) activity only in mitochondria isolated from U87MG human glioma cells. Additionally, exposure of isolated mitochondria to PUFA combinations was associated with a selective increase in the level of reactive oxygen species (ROS), the collapse of the mitochondrial membrane potential, mitochondrial swelling, and cytochrome c liberation. However, these effects were not observed in mitochondria isolated from HEK293 cells [63].

As mentioned above, our experiments based on the administration of marine oils enriched in *ω-3* PUFA did not replicate evidence found by other authors. Moreover, high PUFA doses significantly increased glial mitochondrial activity and, thus, their metabolism and viability. This could be a hallmark of the aggressiveness of this tumoral cell line. GB is the most aggressive and deadliest brain malignancy in adults [62]. Although GB occurs more frequently in elderly people (median age, 64 years), it may present itself at any age, but it is rare in children. GB progression cannot be controlled by surgical resection, radiotherapy, or chemotherapy, resulting in a median survival time of <15 months [64].

Further, astrocytes can endocytose lipid particles from hyperactive neurons, especially those ApoE-positives, and sequester fatty acids into lipid droplets [64]. In addition, it is also well reported that astrocytes are able to break down lipid droplets and feed liberated FA into the mitochondria as fuel for oxidative phosphorylation. During this process, astrocytes upregulate genes involved in energy metabolism [64]. Fatty acid metabolism is coupled between neurons and astrocytes so that fatty acids can then be consumed by oxidative phosphorylation in astrocyte mitochondria to protect neurons from their toxicity during periods of enhanced activity [65]. As glial cells serve a variety of functions as metabolic support for neurons [66], metabolic coordination between neurons and astrocytes is critical for the health of the brain [65]. These metabolic capacities of astrocytes could explain the maintenance or even the increase that we found in the glial spheroids’ metabolism after PUFA supplementation.

In addition, the poor water solubility of DHA and its susceptibility to degradation have been discussed as possible reasons for the necessity of new therapeutic approaches [67,68]. The development of DHA liposomes through a microfluidic system has been proposed to overcome the difficulties of administering *ω-3* PUFA while increasing their bioavailability and maintaining their specific activity for treating GB [68]. The development of ω-liposomes has been suggested to possess several advantages over the classical dietary intake of DHA, which requires a long duration of intake at high doses [67]. The induction of apoptosis markers was significantly more pronounced for DHA liposomes compared to free DHA, potentially attributed to the instability and susceptibility of the free PUFA to oxidative reactions [68].

### 2.4. Effect of Polar Marine Oils on Non-Neural Cell Line Viability

**Figure 11:**
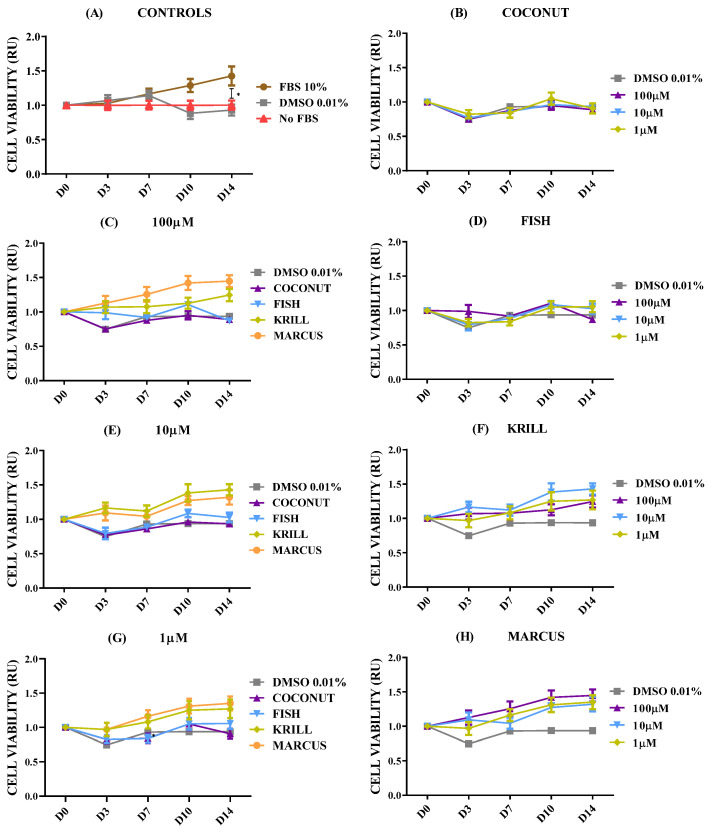
Cell viability relative units (RU) in HEK293T spheroids according to oil types and concentrations. n = 8. (**A**) Control spheroids in FBS 10%, DMSO 0.01%, and no FBS. (**B**) Spheroids in DMSO 0.01% and coconut oil. (**C**) Spheroids in DMSO 0.01% and 100 µM coconut, fish, krill, and squid oils. (**D**) Spheroids in DMSO 0.01% and fish oil. (**E**) Spheroids in DMSO 0.01% and 10 µM coconut, fish, krill, and squid oils. (**F**) Spheroids in DMSO 0.01% and krill oil. (**G**) Spheroids in DMSO 0.01% and 10 µM coconut, fish, krill, and squid oils. (**H**) Spheroids in DMSO 0.01% and squid oil. Only multiple comparisons tests are represented. ANOVA and paired *t*-test are listed in Table S4. (*) *p* ≤ 0.05.

A set of experiments was performed in non-neural cell lines to demonstrate the specificity of the activity of marine oils over NB. Therefore, experiments were set up over spheroids of the human embryonic kidney line. HEK293T cells were able to colonize the hydrogel efficiently, as can be observed in the control spheroids (Figure 11A and Figures S12–S14). Moving out of the central nervous system, *ω-3* PUFA treatment induced an apoptotic effect, which was synergistic with cytotoxic drugs in different tumor cell lines, such as melanoma [69], colon [70], pancreatic and leukemic [71], breast [72], or prostate [73,74,75].

**Figure 12:**
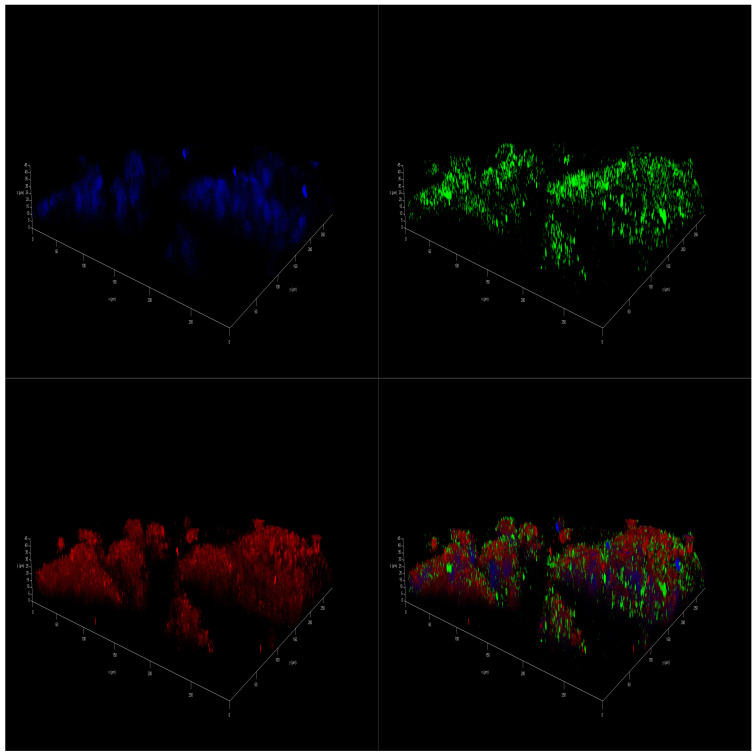
IF 3D images of HEK293T spheroids at day 7 (D14) in culture medium with 100 µM of Krill oil. Spheres are stained with DAPI^TM^ for the nucleus in blue, Alexa 488 for β-actin in green, and MitoTracker^TM^ in red.

No oil treatment decreased cell viability. Treatment with coconut oil (SFA carried as TG) and fish oil (PUFA carried as TG) resulted in spheroids having almost identical metabolic activity curves. When PUFAs were added as PL (krill and squid oils), significant differences led to an increase in cell viability, no matter what concentration was used (Figure 11C,E,G). This positive effect was also seen when mitochondrial activity was stained (Figure 12 and Figure S15). EPA and DHA fatty acids added individually slightly replicated the effect of each oil, without reducing the metabolic rate (Figure S16 and Table S7).

As already discussed in the previous item, exposure of isolated mitochondria from HEK293 cells to PUFA combinations did not have an impact on the mitochondria function [63]. Moreover, supplementation with *ω-3*, *ω-6*, and *ω-9* fatty acids did not affect HEK293 cells’ viability in the range of doses that have demonstrated beneficial effects in earlier studies [76]. None of the marine oils used led to a negative effect on cell viability and metabolic activity; indeed, polar marine oils such as krill and squid oils improved both parameters. Therefore, marine oils did not exert any antiproliferative effect over non-neural and non-tumoral cells, providing some evidence about the safety and innocuousness of the treatments.

### 2.5. Effect of Polar Marine Oils on the Expression of NB Signaling Proteins and miRNAs

**Figure 13:**
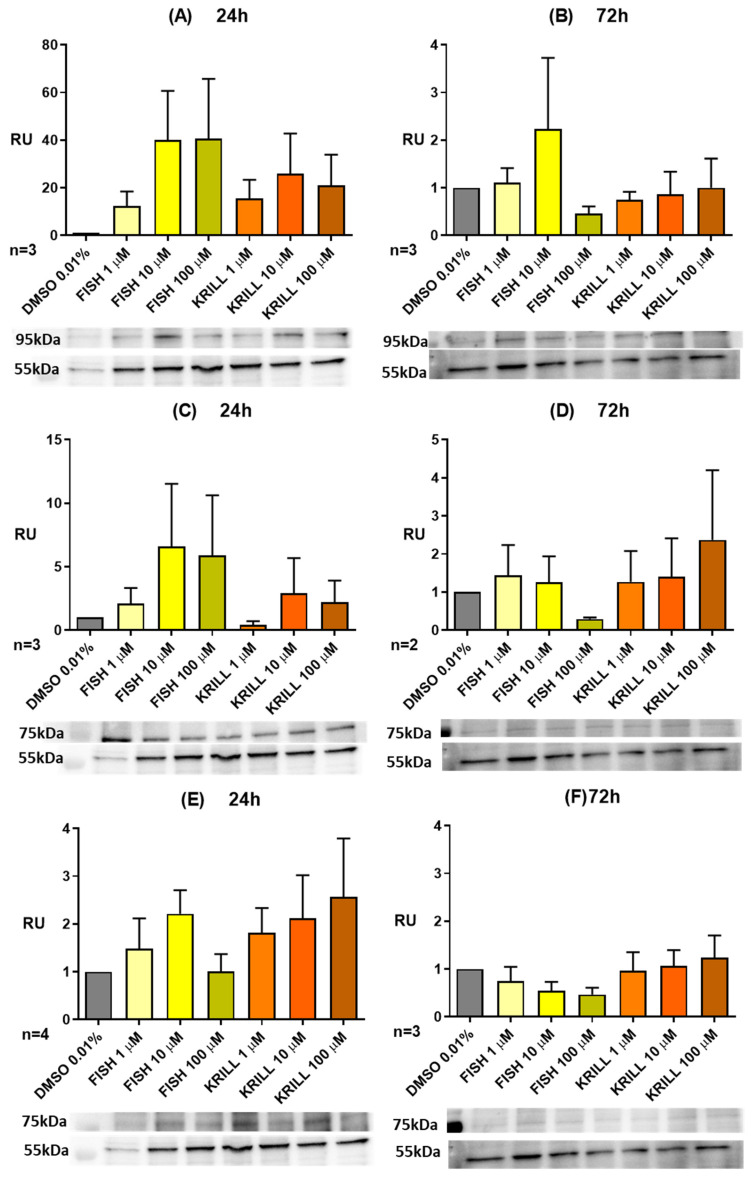
Dose/response protein expression analysis measured by WB. Protein expression was normalized to βIII-tubulin. RU: relative units to DMSO 0.01%. (**A**) Synapsin IIa after 24 h. (**B**) Synapsin IIa after 72 h. (**C**) TrkB after 24 h. (**D**) TrkB after 72 h. (**E**) p75-NGF-R after 24 h. (**F**) p75-NGF-R after 72 h.

A combined protein signaling and miRNA expression profiling study was performed on NB SH-SY5Y to go deeper into the mechanisms behind the time- and concentration-dependent anti-proliferative effect of polar vs. non-polar marine oils on the human NB spheroids. Figure 13 represents the dose/response curves of fish oil and krill oil for protein expression analysis over NB SH-SY5Y cell lines, illustrating the results corresponding to proteins related to neuronal plasticity, viability, and differentiation, such as Synapsin IIa, and neurotrophin receptors TrkB and p75-NGF-R. This increase was dependent on the oil concentration. A remarkable effect was also implied on the basis of the molecular presentation of PUFA since TGs from fish oil were more able than krill oil PLs to induce Synapsin IIa and TrkB expression, whereas p75-NGF-R appears to be more sensitive to PL addition.

Results showed that marine oils induced the expression of these proteins at the early incubation (24 h), but the effects disappeared when treatments were extended to a longer period (72 h). Exposure to *ω-3* PUFA enhanced adult hippocampal neurogenesis, promoted synaptic plasticity by increasing long-term potentiation, and modulated synaptic protein expression to stimulate dendritic arborization [77]. We observed a clear increase in the expression of Synapsin IIa after 24 h of *ω-3* PUFA supplementation. Results follow the same trend as other synaptic vesicle proteins, such as synaptophysin and synaptotagmin-1, which seem to be regulated by *ω-3* PUFA [78]. Deficiencies in *ω-3* significantly reduced their concentration in hippocampal mice synaptosomes, as compared to the *ω-3*-enriched groups [78].

Neurotrophin receptor TrkB had a similar pattern of expression, with a clear induction and then a return to control conditions. Treatment with *ω-3* PUFA-enriched fish oils has been associated with an increase in the protein levels of the TrkB signaling pathway in schizophrenic rat brains [72]. Additionally, DHA significantly increased the phosphorylation levels of the TrkB receptor [79]. DHA also stimulated the expression of TrkB and its agonist brain-derived neurotrophic factor (BDNF). Further, a high-DHA maternal diet increased the mRNA expression of BDNF and TrkB signaling pathways in the fetal brain as gestation progressed [80].

P75-NGF-R promotes neurodegeneration and apoptosis in response to cellular injury caused by oxidative stress [81]. P75-NGF-R expression was inducted, but with less intensity, and, as was previously mentioned, *ω-3* treatments induced a slightly different expression pattern than the previously described proteins. TGs seemed to be more effective in their modulation than PLs. This balance between TrkB and p75-NGF-R has also been reported; EPA supplementation upregulated the expression of the TrKB receptor and its agonist, BDNF, and downregulated the p75 receptor expression in a rodent model of neuroinflammation [82]. In a depression model of olfactory bulbectomized rats, EPA again upregulated the anti-apoptotic BDNF receptor TrkB but suppressed its pro-apoptotic receptor p75-NGF-R [83], suggesting that EPA can elicit neuroprotective effects by modulating neurotrophin signaling [84].

**Figure 14:**
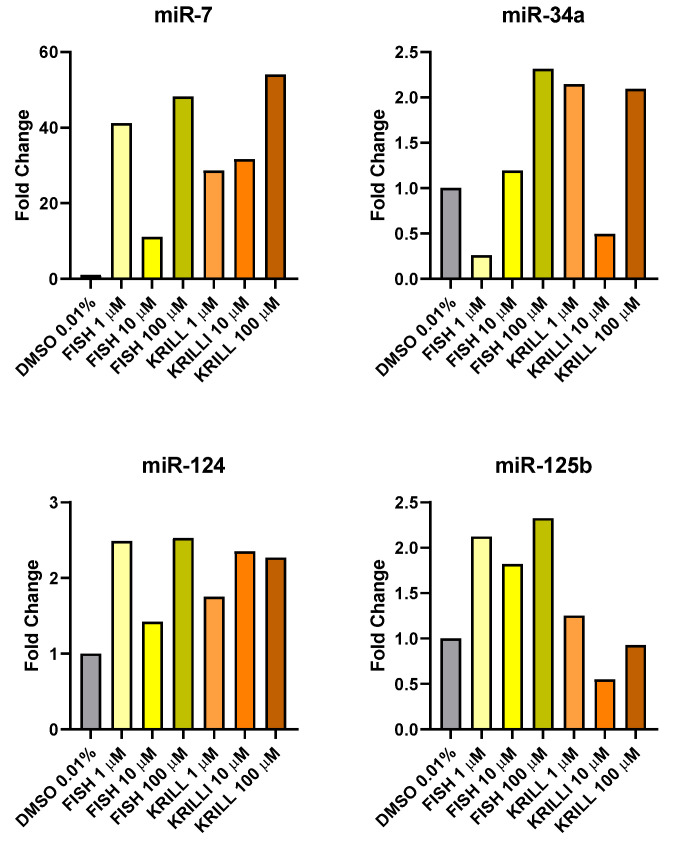
MiR-7, miR-34a, miR-124, and miR-125b fold-change expression compared to control DMSO 0.01% after 48 h of treatment with fish and krill oils at 1, 10, and 100 µM on NB SH-SY5Y cell lines.

The study was complemented with the expression of several miRNAs related to NB apoptosis inductor (mir-7) [85], tumor suppressor (miR-34a) [86] and differentiation promoters (miR-124, miR-125b) [87,88] (Figure 14).

Treatment with both PUFA-enriched oils increased the expression of the miRNA panel, showing the possible contribution of the *ω-3* supplementation to neuronal differentiation and reduction of proliferation in NB. Expression was especially high in the case of apoptosis inductor miR-7. miR-7 and miR-34a expressions were found significantly increased in NB cells after polyphenol treatments that were also able to reduce cell viability in NB [84]. Differences based on the chemical form of PUFA only were manifested in the expression of NB differentiation promoter miR-125b, being double for TG in comparison to PL. Also, with less clarity, miR-34a appears to be more sensible to non-polar than polar oils. *ω-3* PUFA were able to significantly induce miR-34a, enhancing dexamethasone cell sensitivity in multiple myeloma cells [89]. DHA was also able to induce neuroprotection in contusion injury, via miR-124-dependent reduction in the phagocytic response of microglia [90].

miRNAs play several key roles in brain neurodevelopment but also normal function. PUFA contributes epigenetically to brain development and neurogenesis. *ω-3* PUFA were able to modulate several miRNAs involved in the regulation of post-synaptic density proteins being able to normalize their expression after neurotoxicity events [91]. Moreover, high-3 PUFA-enriched diets epigenetically caused hypermethylation of several genes associated with neurodegenerative diseases, enriching cytoskeletal development networks and promoting the formation of neuronal precursors [92]. Krill oil is able to modulate the expression of miRNA genes related to synaptic activity and/or neurodegeneration such as miR-148a-3p, -370-3r, -379-5p, -99a-5p and let-5f-5p [93].

## 3. Materials and Methods

### 3.1. Fatty Acid Composition, Fractional Characterization, and Use for Cell Culture of the Tested Oils

To determine the fatty acid composition of the samples, 0.6 mg of lipid was methylated according to Lepage and Roy [94]. The fatty acid nonadecanoic acid (Merk, Darmstadt, Germany) was used as an internal standard. The FAMEs were analyzed by gas chromatography GC-FID (PerkinElmer Clarus 500 chromatograph, Madrid, Spain), using a SP-2330 fused silica capillary column (30 m × 0.25 mm i.d., 0.20 µm; (Supelco Inc., Bellefonte, PA, USA). Sample injection volume was 1 µL. Oven temperature program was 140 °C for 0.00 min and 1.0 °C/min to 205 °C, hold for 0.00 min. Carrier gas flow rate was 5.2 mL/min. Injector temperature was 275 °C. The quantitative response of the equipment was checked with a GC quantitative standard (FAME Mix, Supelco, Inc., Bellefonte, PA, USA).

Fractional composition of the four oils was compared using thin-layer chromatography on silica gel plates, using mobile phases of n-hexane ≥ 97% (VWR Radnor, PA, USA), diethyl ether (Sigma-Aldrich, St. Louis, MO, USA), and acetic acid (VWR Radnor, PA, USA) (80:20:2). Image J 1.54K software (National Institutes of Health, Bethesda, MD, USA) was used to analyze the blot images acquired and densitometric band quantification. Figure S17 shows thin-layer chromatography of lipid classes identified.

EPA and DHA fatty acid alone were kindly given by Prof. Thierry Durand from Bioactive Lipids Syntheses group (IBMM, Montpellier, France).

Oils were dissolved in DMSO (D2650, Sigma-Aldrich, St. Louis, MO, USA) to make a 1 M stock working solution. To avoid oil aggregation and availability problems for cells, stock solutions were warmed at 37 °C in a water bath, turning solid oils from preserved at 4 °C to liquid. Spheroids were treated at 1, 10, and 100 µM in the culture medium without FBS. DMSO 0.01% was used as a control treatment.

### 3.2. Cell Lines Culture

SH-SY5Y (NB), U251 (GB), and HEK293T cell lines (ATCC, Manassas, VA, USA) were cultured in DMEM/F12 (105650-018, Gibco, ThermoFisher, Waltham, MA, USA) and DMEM with GlutaMAX^TM^ (10569-010, Gibco, ThermoFisher, Waltham, MA, USA), and supplemented with 10% Fetal Bovine Serum (FBS) Heat-Inactivated (S181BH-500, Biowest, Nuaillé, Francia) and 1% Penicillin/Streptomycin (15140-122, Gibco, ThermoFisher Waltham, MA, USA).

### 3.3. Alginate–Carboxymethyl Chitosan Spheroids

Sodium alginate (SA) (2.5% *w*/*v*) (W201502, Sigma-Aldrich, St. Louis, MO, USA) and N(Carboxymethyl) chitosan (CMC) (2.5% *w*/*v*) (sc-358091, Santa Cruz, Dallas, TX, USA) were dissolved in HEPES buffer (0.025 M) (15630080, ThermoFisher, Waltham, MA, USA). Cells were encapsulated in the alginate–CMC hydrogel with a final 2% concentration of SA and CMC. Hydrogel-encapsulated cells were then gelificated in calcium chloride (CaCl_2_) dihydrate (223506, Sigma-Aldrich, St. Louis, MO, USA) as a 0.1 M aqueous solution to induce cationic crosslinking of alginate. After hydrogel gelation, the excess of CaCl_2_ solution was removed, and spheroids were washed 3 times with fresh culture medium.

### 3.4. Cell Viability and Metabolism Assay

AlamarBlue^TM^ cell viability reagent (DAL1100, Invitrogen, ThermoFisher, Waltham, MA, USA) has resazurin as the active ingredient, which is non-toxic, blue in color, and non-fluorescent. Upon entering live cells, the cellular reducing environment reduces resazurin to resorufin, a compound that is red and highly fluorescent. Therefore, cell viability can be detected using a FLUOstar Omega microplates reader (BMG Labtech, Ortenberg, Germany). Spheroids were incubated in the presence of AlamarBlue^TM^ 1:10 in a culture medium for 3 h and protected from direct light. After incubation, the medium was removed, and fluorescence/absorbance was measured.

For fluorescence, excitation wavelength of 560 nm and an emission of 590 nm were used. Cell viability was calculated by using resorufin fluorescence of each day normalized to the first day. For absorbance, the reagent was measured for 570 nm and 600 nm wavelengths before (t0) and after (tx) incubation. The reduction percentage was calculated by using molar extinction coefficients for oxidized and reduced forms of AlamarBlue^TM^ in the following formula (Figure S18) and data (Table S8).

### 3.5. Immunofluorescence Staining

MitoTracker^TM^ Red CMXRos (9082, Invitrogen, Fisher Scientific, Hampton, NH, USA) was used for mitochondrial labeling. Cells were incubated with MitoTracker^TM^ at 100nM diluted in culture medium for 45 min at 37 °C. After incubation, we proceeded with fixation and immunostaining.

Cell-laden alginate–chitosan hydrogels were fixed with ice-cold methanol for 15 min. Fixed spheroids were washed with Dulbecco’s phosphate-buffered saline (DPBS) with Ca^2+^ and Mg^2+^ (14040-091, Gibco, ThermoFisher, Waltham, MA, USA) 3 times for 5 min. Permeabilization was performed with Triton X-100 0.1% for 15 min and then washed again with DPBS 3 times for 5 min. Next, 5% bovine serum albumin (BSA) blocking was performed for 60 min and washed with PBS. Then, spheroids were incubated for 90 min with primary antibody. After another DPBS wash, a secondary antibody was incubated for 45 min. Finally, DAPI (D9542, Sigma-Aldrich, St. Louis, MO, USA) staining was performed for 5 min, and then spheres were washed with DPBS and left in this buffer until imaging in a STELLARIS 8 Laser scanning Confocal Microscope from Leica Microsystems.

Primary antibodies used were anti-Tubulin beta 3 mouse antibody 1:1000 (212-301-E55; Rockland, Pottstown, PA, USA), anti-GFAP mouse monoclonal antibody 1:1000 (200-301-W55; Rockland, Pottstown, PA, USA), and anti-Beta Actin rabbit antibody 1:1000 (600-401-886; Rockland, Pottstown, PA, USA). Secondary antibodies used were Alexa Fluor^TM^ 488 donkey anti-mouse IgG (H + L) 1:250 (A21202; Invitrogen, ThermoFisher, Waltham, MA, USA) and Alexa Fluor^TM^ 488 donkey anti-rabbit IgG (H + L) 1:250 (A21206; Invitrogen, ThermoFisher, Waltham, MA, USA).

### 3.6. Western Blot

Proteins from cell cultures were measured using a BCA assay (Piercce Chemicals, Rockford, IL, USA). Protein samples were mixed with 2 × volume of Laemmli buffer 2× (Bio-Rad, Hercules, CA, USA) (950 µL of Laemmli buffer and 50 µL of β-mercaptoethanol) and lysis buffer PIK (Table S9) and then boiled at 95 °C for 5 min. Fraction samples were loaded in 8% Bis-Tris polyacrylamide gels, and electrophoresis was performed in a Power Pac^TM^ universal power supply (Bio-Rad, Hercules, CA, USA) at 80 V for 30 min, and at 120 V for another 90 min. The proteins were immediately transferred to polyvinylidene difluoride membranes (Immun-Blot^®^ polyvinylidene fluoride membrane, Bio-Rad, Hercules, CA, USA) contained in a PowerPac^TM^ universal power supply (Bio-Rad, Hercules, CA, USA) set at 0.25 A for 60 min (for two gels). The membranes were blocked with 5% milk powder in a TBST for 20 min and washed three times with the same TBST solution. The membranes were incubated overnight, with stirring, at 4 °C, with a primary antibody listed in Table S10.

After washing the membranes three times with TBST, we incubated them with appropriate rabbit or mouse secondary antibody 1:10,000 (GE Healthcare Life Sciences, Coventry, UK), stirring for 60 min. The membranes were then washed again twice with TBST and once with TBS. The ChemiDoc XRS+ system (Bio-Rad, Hercules, CA, USA) was then used to analyze the chemiluminescence of the membranes with the ECL^TM^ Prime Western Blotting System (Sigma-Aldrich, St. Louis, MO, USA). Image Lab 6.0 software (Bio-Rad, Hercules, CA, USA) was used to analyze the blot images acquired and densitometric band quantification. Western blot normalization was performed individually using β-III-tubulin for neurons (Table S10), converting results into percentages expressed in relative units.

### 3.7. RNA Extraction

Total RNA was extracted and purified from well cultures using a miRNeasy Mini Kit (Qiagen, Hilden, Germany), following the manufacturer’s protocol. BioDrop µLite 7141 V1.0.3 was used to determine 260/280 and 260/230 purity ratios. The quantity and integrity of the RNA were assessed using an RNA 6000 Nano Kit, and the % of the miRNA fraction was measured using a Small RNA Kit, on a 2100 Bioanalyzer (Agilent Technologies, Santa Clara, CA, USA).

### 3.8. Reverse Transcription and Real Time-qPCR

A total of 10 ng of total isolated RNA was prepared for cDNA synthesis by using TaqMan^®^ Advanced miRNA cDNA Synthesis Kit. cDNA samples were then diluted 1:10 to perform RT-qPCR. TaqMan^®^ Universal Master Mix II, no UNG, and TaqMan^®^ Advanced miRNA Assays (20X) for has-miR-7, has-miR-34a, has-miR-124, and has-miR-125b were used. Both procedures were performed following the manufacturer’s guidelines.

### 3.9. Statistical Analysis

Long-time treatment data analysis was performed via normalization of each sphere toward its day 0 (D0) measurement. Next, each sphere was referred to its blank control (culture medium without FBS). Data were plotted as mean and standard error using GraphPad Prism 8.0.1 software (GraphPad Software Inc., San Diego, CA, USA). Normality was assessed with a Normality and Lognormality test. Two-way ANOVA or Kruskal–Wallis test followed by Tukey’s or Dunn’s multiple comparisons post hoc test was used to determine statistical differences across the groups. A group-to-group comparison was made by paired or Wilcoxon *t*-test. *p*-Value ≤ 0.05 was considered statistically significant.

## 4. Conclusions

*ω-3* PUFA (i.e., EPA or DHA) oils can reduce NB 3D spheroid viability significantly to very low levels, associated with a reduction in the metabolic activity. Although these results do not provide definitive evidence of mitochondrial dysfunction, they suggest that this may be a mechanism by which *ω-3* PUFAs mediate their antitumor effects.

Marine oils exhibited time- and concentration-dependent anti-proliferative effects on the human NB SH-SY5Y spheroids, but they had no effect on GB U251 cells and a positive proliferative effect on non-neural and non-tumorigenic HEK293T cell line. We hypothesize that the divergences between NB and GB spheroid response might be due to their different physiological and energetical characteristics.

Polar and non-polar *ω-3*-enriched oils have this specific effect on neuroblastoma. However, cell response to non-polar oils was higher at lower concentrations. Thus, the chemical presentation in which fatty acids are supplemented may be an interesting factor for these selective antiproliferative properties that we are presenting.

Sustainable and polar marine oil coming from squid by-products show bioactive antiproliferative characteristics similar to commercial polar krill oil, introducing a new potential use for fishery discards.

Supplementation with *ω-3* fatty acid may contribute to inducing the expression of proteins and miRNA involved in neuronal differentiation. However, this protective outcome is eventually overcome, as long-time supplementation modulates neural metabolism and cell proliferation.

## Figures and Tables

**Table 1:** Fatty acid composition for coconut, fish, krill, and squid oils (mmol FA/100 g of oil).

Fatty Acid	Coconut	Fish	Krill	Squid
**8:0**	30.59	-	-	-
**10:0**	55.95	-	-	-
**11:0**	44.29	-	-	-
**12:0**	270.03	-	-	-
**14:0**	87.59	26.69	36.56	5.07
**15:0**	-	1.92	1.38	0.70
**16:0**	34.02	61.85	57.59	28.98
**16:1** ***ω*** ***7***	-	28.35	16.50	2.62
**17:0**	-	2.70	5.49	1.10
**18:0**	11.11	15.13	3.71	4.42
**18:1** ***ω*** ***9***	17.86	33.32	27.69	5.47
**18:1** ***ω*** ***7***	-	10.39	15.96	3.15
**18:2** ***ω*** ***6***	2.59	4.05	4.66	0.88
**20:0**	-	1.81	0.25	0.22
**18:3** ***ω*** ***3***	-	1.72	3.95	0.67
**20:1** **ω** ***9***	-	2.48	1.66	5.33
**18:4** ***ω*** ***3***	-	5.39	10.69	2.13
**20:2** ***ω*** ***6***	-	0.68	0.29	0.50
**20:3** ***ω*** ***6***	-	0.45	0.21	0.00
**20:4** ***ω*** ***6***	-	3.25	0.99	2.43
**22:1** ***ω*** ***11***	-	1.25	0.34	0.89
**22:1** ***ω*** ***9***	-	0.42	1.20	0.66
**20:4** ***ω*** ***3***	-	1.83	0.95	0.46
**20:5*ω*3 (EPA)**	-	45.27	36.24	19.54
**22:4** ***ω*** ***6***	-	1.98	1.15	0.31
**24:1** ***ω*** ***9***	-	0.64	0.25	0.78
**22:5** ***ω*** ***6***	-	1.14	0.13	0.36
**22:5** ***ω*** ***3***	-	4.52	0.90	0.52
**22:6*ω*3 (DHA)**	-	24.30	19.58	32.78
**total mmol/100 g**	554.03	281.54	248.31	119.95
**total mmol/g**	5.54	2.82	2.48	1.20

**Table 2:** Percentage (%) of lipid classes composition for coconut, fish, krill, and squid oils.

Lipid Classes	Coconut	Fish	Krill	Squid
**PL ^1^**	-	-	32.31	43.36
**O-A-DAG ^2^**	-	1.26	1.19	0.84
**MG ^3^**	-	-	-	3.43
**DG ^4^**	-	t ^9^	t ^9^	-
**CH ^5^**	-	t ^9^	6.96	11.13
**FFA ^6^**	-	-	19.18	31.97
**TG ^7^**	99	95	40.37	t ^9^
**CH-ES ^8^**	-	-	-	9.27

^1^ Phospholipids; ^2^ o-alkyl-diacylglycerols; ^3^ monoacylglycerols; ^4^ diacylglycerols; ^5^ cholesterol; ^6^ free fatty acids; ^7^ triacylglycerols; ^8^ cholesteryl-esters; ^9^ trace content.

## Data Availability

Data are contained within the article or Supplementary Materials; further inquiries can be directed toward the corresponding author.

## Supplementary Materials

The following supporting information can be downloaded at https://www.mdpi.com/article/10.3390/md23070268/s1, Figure S1: IF 3D images of SH-SY5Y spheroids on day 0 (D0) of being in culture medium with 100% FBS; Figure S2: IF 3D images of SH-SY5Y spheroids on day 14 (D140) of being in culture medium with 100% FBS; Figure S3: IF 3D images of SH-SY5Y spheroids on day 14 (D14) of being in culture medium with 0.01% DMSO; Figure S4: IF 3D images of SH-SY5Y spheroids on day 14 (D14) of being in culture medium with 100 µM of coconut oil; Figure S5: IF 3D images of SH-SY5Y spheroids on day 14 (D14) of being in culture medium with 100 µM of EPA; Figure S6: IF 3D images of SH-SY5Y spheroids on day 14 (D14) of being in culture medium with 100 µM of DHA; Figure S7: Cell viability relative units (RU) in U251 spheroids according to isolated EPA and DHA fatty acids; Figure S8: IF 3D images of U251 spheroids on day 0 (D0) of being in culture medium with 100% FBS; Figure S9: IF 3D images of U251 spheroids on day 14 (D14) of being in culture medium with 100% FBS; Figure S10: IF 3D images of U251 spheroids on day 14 (D14) of being in culture medium with 100 µM of coconut oil; Figure S11: IF 3D images of U251 spheroids on day 14 (D14) of being in culture medium with 100 µM of squid oil; Figure S12: IF 3D images of HEK293T spheroids on day 0 (D0) of being in culture medium with 100% FBS; Figure S13: IF 3D images of HEK293T spheroids on day 14 (D14) of being in culture medium with 100% FBS; Figure S14: IF 3D images of HEK293T spheroids on day 14 (D14) of being in culture medium with 0.01% DMSO; Figure S15: IF 3D images of HEK293T spheroids on day 14 (D14) of being in culture medium with 100 µM of coconut oil; Figure S16: Cell viability relative units (RU) in HEK293T spheroids according to isolated EPA and DHA fatty acids; Figure S17: Thin-Layer Chromatography for lipid classes identification; Figure S18: % of reduction formula for AlamarBlue^TM^ assay; Table S1: 2-way ANOVA and Paired *t*-test of Figure 2; Table S2: 2-way ANOVA and Paired *t*-test of Figure 5; Table S3: 2-way ANOVA and Paired *t*-test of Figure 7; Table S4: 2-way ANOVA and Paired *t*-test of Figure 8; Table S5: 2-way ANOVA and Paired *t*-test of Figure S7; Table S6: 2-way ANOVA and Paired *t*-test of Figure 11; Table S7: 2-way ANOVA and Paired *t*-test of Figure S16; Table S8: Molar Extinction Coefficients (ε) for AlamarBlue^TM^; Table S9: Lysis buffer (PIK) composition for 200 mL; Table S10: Primary antibodies used in Western Blot.
