# Supplementary material for: Selective Antiproliferative Effects of Marine Oils on Neuroblastoma Cells in 3D Cultures
Source: Mar Drugs. 2025 Jun 26;23(7):268. doi: 10.3390/md23070268 (PMC12300571; doi:10.3390/md23070268)
Supplement: Supplementary file 1 [file marinedrugs-23-00268-s001.zip › Supplementary Tables and Figure S18.pdf]

Table S1. 2-way ANOVA and Paired t-test of Figure 2: Cell viability relative units (RU) in SH-SY5Y spheroids according to oil types and concentrations. n=8.

Subfigure	Statistical Analysis	Variability / Comparison	Significance
A) Control spheroids in FBS 10%, DMSO 0.01%, and No FBS.	2-way ANOVA	Interaction	****
		Time	****
		Treatment	****
	Paired *t*-test	FBS - DMSO	***
B) Spheroids in DMSO 0.01% and Coconut oil.	2-way ANOVA	Interaction	ns
		Time	****
		Treatment	ns
	Paired *t*-test	100μM-1μM	#
		10μM-1μM	*
		1μM-DMSO	#
C) Spheroids in DMSO 0.01% and 100μM Coconut, Fish, and Krill oils.	2-way ANOVA	Interaction	****
		Time	****
		Treatment	****
	Paired *t*-test	FISH-DMSO	*
		FISH-COCONUT	*
		KRILL-DMSO	*
		KRILL-COCONUT	*
D) Spheroids in DMSO 0.01% and Fish oil.	2-way ANOVA	Interaction	****.
		Time	****.
		Treatment	****.
	Paired *t*-test	100μM-DMSO	*
		10μM-DMSO	*
		10μM-1μM	#
		1μM-DMSO	*
E) Spheroids in DMSO 0.01% and 10μM Coconut, Fish, and Krill oils.	2-way ANOVA	Interaction	****
		Time	****
		Treatment	****
	Paired *t*-test	FISH-DMSO	*
		FISH-COCONUT	*
		KRILL-FISH	*
F) Spheroids in DMSO 0.01% and Krill oil	2-way ANOVA	Interaction	****
		Time	****
		Treatment	****
	Paired *t*-test	-	ns
G) Spheroids in DMSO 0.01% and 1μM Coconut, Fish, and Krill oils.	2-way ANOVA	Interaction	****
		Time	****
		Treatment	****
	Paired *t*-test	FISH-DMSO	*
		FISH-COCONUT	*
		KRILL-FISH	*

(****) p≤0.0001; (***) p≤0.001; (**) p≤0.01; (*) p≤0.05; (#) p≤0.1.

Table S2. 2-way ANOVA and Paired t-test of Figure 5: Cell viability relative units (RU) in SH-SY5Y spheroids according to Squid oil and concentrations. n=8.

Subfigure	Statistical Analysis	Variability / Comparison	Significance
A) Spheroids in DMSO 0.01% and Squid oil.	2-way ANOVA	Interaction	****
		Time	****
		Treatment	****
	Paired *t*-test	100μM-DMSO	#
		100μM-10μM	*
		100μM-1μM	*
B) Spheroids in DMSO 0.01% and 100μM Coconut, Fish, Krill, and Squid oils.	2-way ANOVA	Interaction	ns
		Time	****
		Treatment	ns
	Paired *t*-test	-	ns
C) Spheroids in DMSO 0.01% and 10μM Coconut, Fish, Krill, and Squid oils.	2-way ANOVA	Interaction	****
		Time	****
		Treatment	****
	Paired *t*-test	-	ns
D) Spheroids in DMSO 0.01% and 1μM Coconut, Fish, Krill, and Squid oils.	2-way ANOVA	Interaction	****.
		Time	****.
		Treatment	****.
	Paired *t*-test	-	ns

(****) p≤0.0001; (***) p≤0.001; (**) p≤0.01; (*) p≤0.05; (#) p≤0.1.

Table S3. 2-way ANOVA and Paired t-test of Figure 7: Cell viability relative units (RU) in SH-SY5Y spheroids according to EPA and DHA. n=8.

Subfigure	Statistical Analysis	Variability / Comparison	Significance
A) Spheroids in DMSO 0.01% and EPA.	2-way ANOVA	Interaction	****
		Time	ns
		Treatment	*
	Paired *t*-test	100μM-DMSO	*
		100μM-10μM	*
		100μM-1μM	*
		10μM-1μM	*
B) Spheroids in DMSO 0.01% and DHA	2-way ANOVA	Interaction	****
		Time	ns
		Treatment	*
	Paired *t*-test	100μM-DMSO	*
		100μM-10μM	*
		100μM-1μM	*
		10μM-DMSO	*
		10μM-1μM	*
		1μM-DMSO	*

(****) p≤0.0001; (***) p≤0.001; (**) p≤0.01; (*) p≤0.05; (#) p≤0.1.

Table S4. 2-way ANOVA and Paired t-test of Figure 8: Cell viability relative units (RU) in U251 spheroids according to oil types and concentrations. n=8.

Subfigure	Statistical Analysis	Variability / Comparison	Significance
A) Control spheroids in FBS 10%, DMSO 0.01%, and No FBS.	2-way ANOVA	Interaction	****
		Time	****
		Treatment	****
	Paired *t*-test	-	ns
B) Spheroids in DMSO 0.01% and Coconut oil.	2-way ANOVA	Interaction	ns
		Time	****
		Treatment	ns
	Paired *t*-test	100μM-DMSO	#
		100μM-10μM	#
C) Spheroids in DMSO 0.01% and 100μM Coconut, Fish, and Krill oils.	2-way ANOVA	Interaction	****
		Time	****
		Treatment	****
	Paired *t*-test	COCONUT-DMSO	#
		FISH-COCONUT	#
		KRILL-COCONUT	#
		SQUID-COCONUT	#
		SQUID-DMSO	*
		SQUID-COCONUT	*
		SQUID-FISH	*
D) Spheroids in DMSO 0.01% and Fish oil.	2-way ANOVA	Interaction	****.
		Time	****.
		Treatment	****.
	Paired *t*-test	-	ns
E) Spheroids in DMSO 0.01% and 10μM Coconut, Fish, and Krill oils.	2-way ANOVA	Interaction	****
		Time	****
		Treatment	ns
	Paired *t*-test	-	ns
F) Spheroids in DMSO 0.01% and Krill oil	2-way ANOVA	Interaction	****
		Time	****
		Treatment	****
	Paired *t*-test	100μM-DMSO	#
		100μM-10μM	*
		10μM-1μM	#
G) Spheroids in DMSO 0.01% and 1μM Coconut, Fish, and Krill oils.	2-way ANOVA	Interaction	****
		Time	****
		Treatment	ns
	Paired *t*-test	-	ns
H) Spheroids in DMSO 0.01% and Squid oil	2-way ANOVA	Interaction	****
		Time	****
		Treatment	**
	Paired *t*-test	100μM-DMSO	*
		100μM-10μM	*
		100μM-1μM	*

(****) p≤0.0001; (***) p≤0.001; (**) p≤0.01; (*) p≤0.05; (#) p≤0.1.

Table S5. 2-way ANOVA and Paired t-test of Figure S7: Cell viability relative units (RU) in U251 spheroids according to isolated EPA and DHA fatty acids. n=8.

Subfigure	Statistical Analysis	Variability / Comparison	Significance
A) Spheroids in DMSO 0.01% and EPA fatty acid.	2-way ANOVA	Interaction	****
		Time	ns
		Treatment	*
	Paired *t*-test	100μM-DMSO	#
		100μM-10μM	*
		100μM-1μM	*
B) Spheroids in DMSO 0.01% and DHA fatty acid.	2-way ANOVA	Interaction	****
		Time	ns
		Treatment	*
	Paired *t*-test	100μM-DMSO	#

(****) p≤0.0001; (***) p≤0.001; (**) p≤0.01; (*) p≤0.05; (#) p≤0.1.

Table S6. 2-way ANOVA and Paired t-test of Figure 11: Cell viability relative units (RU) in HEK293T spheroids according to oil types and concentrations. n=8.

Subfigure	Statistical Analysis	Variability / Comparison	Significance
A) Control spheroids in FBS 10%, DMSO 0.01%, and No FBS.	2-way ANOVA	Interaction	****
		Time	ns
		Treatment	ns
	Paired *t*-test	-	ns
B) Spheroids in DMSO 0.01% and Coconut oil.	2-way ANOVA	Interaction	****
		Time	****
		Treatment	****
	Paired *t*-test	-	ns
C) Spheroids in DMSO 0.01% and 100μM Coconut, Fish, and Krill oils.	2-way ANOVA	Interaction	****
		Time	****
		Treatment	****
	Paired *t*-test	KRILL-DMSO	*
		KRILL-COCONUT	*
		KRILL-SQUID	*
		SQUID-DMSO	*
		SQUID-COCONUT	*
		SQUID-FISH	*
D) Spheroids in DMSO 0.01% and Fish oil.	2-way ANOVA	Interaction	****.
		Time	****.
		Treatment	****.
	Paired *t*-test	-	ns
E) Spheroids in DMSO 0.01% and 10μM Coconut, Fish, and Krill oils.	2-way ANOVA	Interaction	****
		Time	****
		Treatment	ns
	Paired *t*-test	FISH-COCONUT	#
		FISH-KRILL	*
		FISH-SQUID	*
		KRILL-DMSO	*

		KRILL-COCONUT	*
		KRILL-SQUID	*
		SQUID-DMSO	*
		SQUID-COCONUT	*
F) Spheroids in DMSO 0.01% and Krill oil	2-way ANOVA	Interaction	****
		Time	****
		Treatment	****
	Paired *t*-test	100μM-DMSO	*
		100μM-10μM	#
		10μM-DMSO	*
		10μM-1μM	*
		1μM-DMSO	*
G) Spheroids in DMSO 0.01% and 1μM Coconut, Fish, and Krill oils.	2-way ANOVA	Interaction	****
		Time	****
		Treatment	ns
	Paired *t*-test	FISH-KRILL	*
		FISH-SQUID	*
		KRILL-DMSO	*
		KRILL-COCONUT	*
		KRILL-SQUID	#
		SQUID-DMSO	*
		SQUID-COCONUT	*
H) Spheroids in DMSO 0.01% and Squid oil	2-way ANOVA	Interaction	****
		Time	****
		Treatment	**
	Paired *t*-test	100μM-DMSO	*
		100μM-10μM	*
		10μM-D	*
		10μM-1μM	#
		1μM-DMSO	*

(****) p≤0.0001; (***) p≤0.001; (**) p≤0.01; (*) p≤0.05; (#) p≤0.1.

Table S7. 2-way ANOVA and Paired t-test of Figure S16: Cell viability relative units (RU) in HEK293T spheroids according to isolated EPA and DHA fatty acids. n=8.

Subfigure	Statistical Analysis	Variability / Comparison	Significance
A) Spheroids in DMSO 0.01% and EPA fatty acid.	2-way ANOVA	Interaction	****
		Time	ns
		Treatment	*
	Paired *t*-test	100μM-1μM	#
		1μM-DMSO	*
B) Spheroids in DMSO 0.01% and DHA fatty acid.	2-way ANOVA	Interaction	****
		Time	ns
		Treatment	*
	Paired *t*-test	100μM-DMSO	#
		100μM-10μM	#
		100μM-1μM	*

(****) p≤0.0001; (***) p≤0.001; (**) p≤0.01; (*) p≤0.05; (#) p≤0.1.

Table S8. Molar Extinction Coefficients (ε) for AlamarBlue™.

Wavelength	Origin	Type
570 nm	155,677	80,586
600 nm	14,652	117,216

Table S9. Lysis buffer (PIK) composition for 200mL.

Reagent	Amount	Final Concentration	Manufacturer
NaCl	1.75 g	150 mM	Sigma-Aldrich, St.Louis, MO, USA
Trizma® base	0.485 g	50 mM	Sigma-Aldrich, St.Louis, MO, USA
EDTA 5M	2 mL	5 mM	PanReac AppliChem, ITW Reagents, Barcelona, Spain
Distilled H2O	100 mL	-	-
HCL	Adjust pH=7.4		Sigma-Aldrich, St.Louis, MO, USA
Glycerine	20 mL	10%	Sigma-Aldrich, St.Louis, MO, USA
NP-40	2 mL	1%	Calbiochem, Merk Milipore, MA, USA
Distilled H2O	Complete to Final volume 200mL		-

Table S10. Primary antibodies used in Western Blot.

Antibody	Origin	Type	Trading house	Concentration
Anti-Synapsin IIa (H-75)	Rabbit	Polyclonal	Santa Cruz	1:4000
Anti-TrkB	Rabbit	Polyclonal	Abcam	1:100
Anti-p75-NGF-Receptor	Rabbit	Polyclonal	Abcam	1:1000
Anti-βIII-tubulin	Mouse	Monoclonal	Rockland	1:10000

$$\%Reduction = \frac{(\varepsilon_{OX\_600nm} \times A_{570nm\_tx} - \varepsilon_{OX\_570nm} \times A_{600nm\_tx})}{(\varepsilon_{RED\_570nm} \times A_{600nm\_t0} - \varepsilon_{RED\_600nm} \times A_{570nm\_t0})}$$

Figure S18: % of reduction formula for AlamarBlue™ assay.
