# Supplementary figures and images for: Selective Antiproliferative Effects of Marine Oils on Neuroblastoma Cells in 3D Cultures
Source: Mar Drugs. 2025 Jun 26;23(7):268. doi: 10.3390/md23070268 (PMC12300571; doi:10.3390/md23070268)

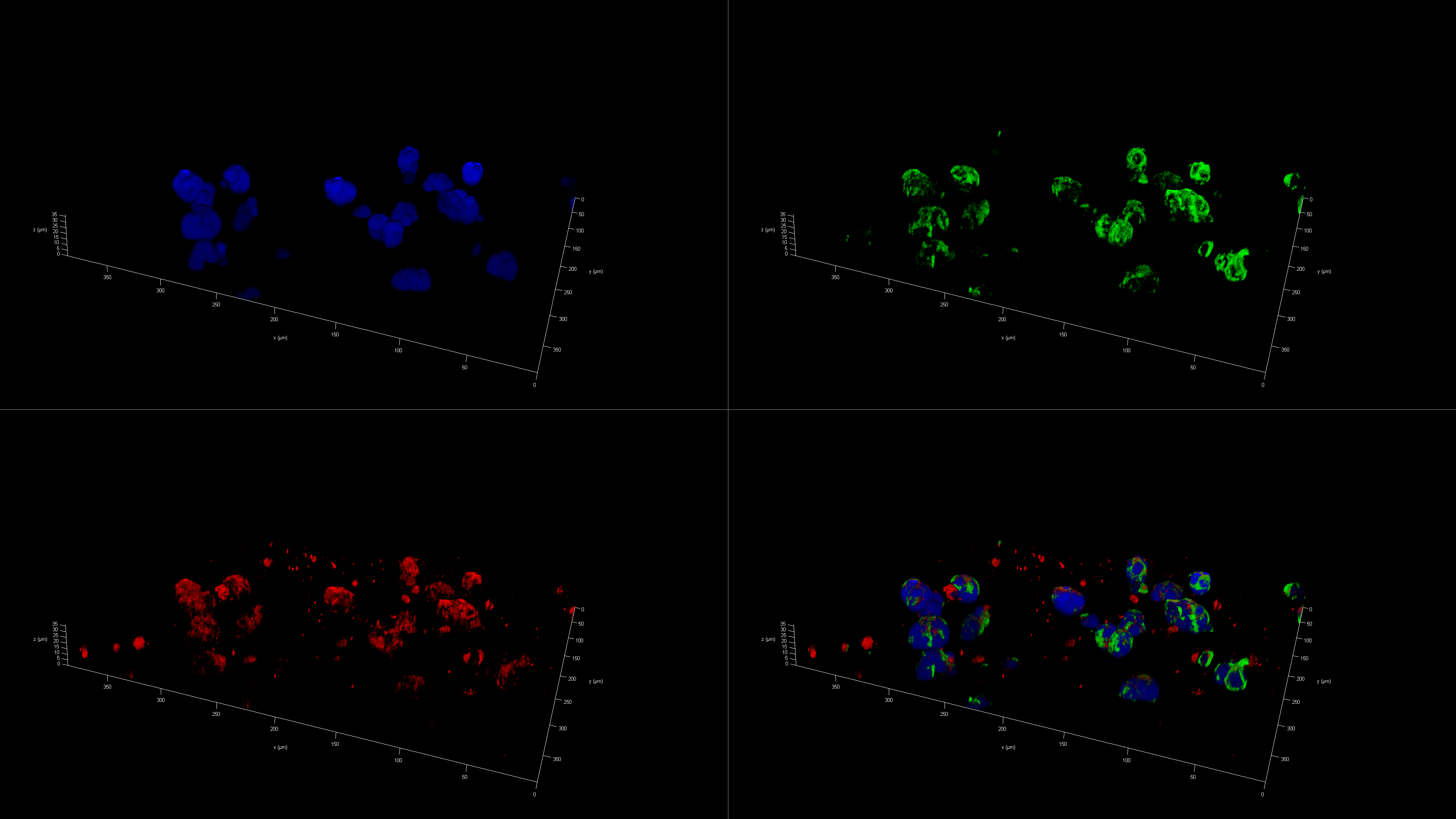

Supplement: Supplementary file 1 [file marinedrugs-23-00268-s001.zip › Figure S1.tif]

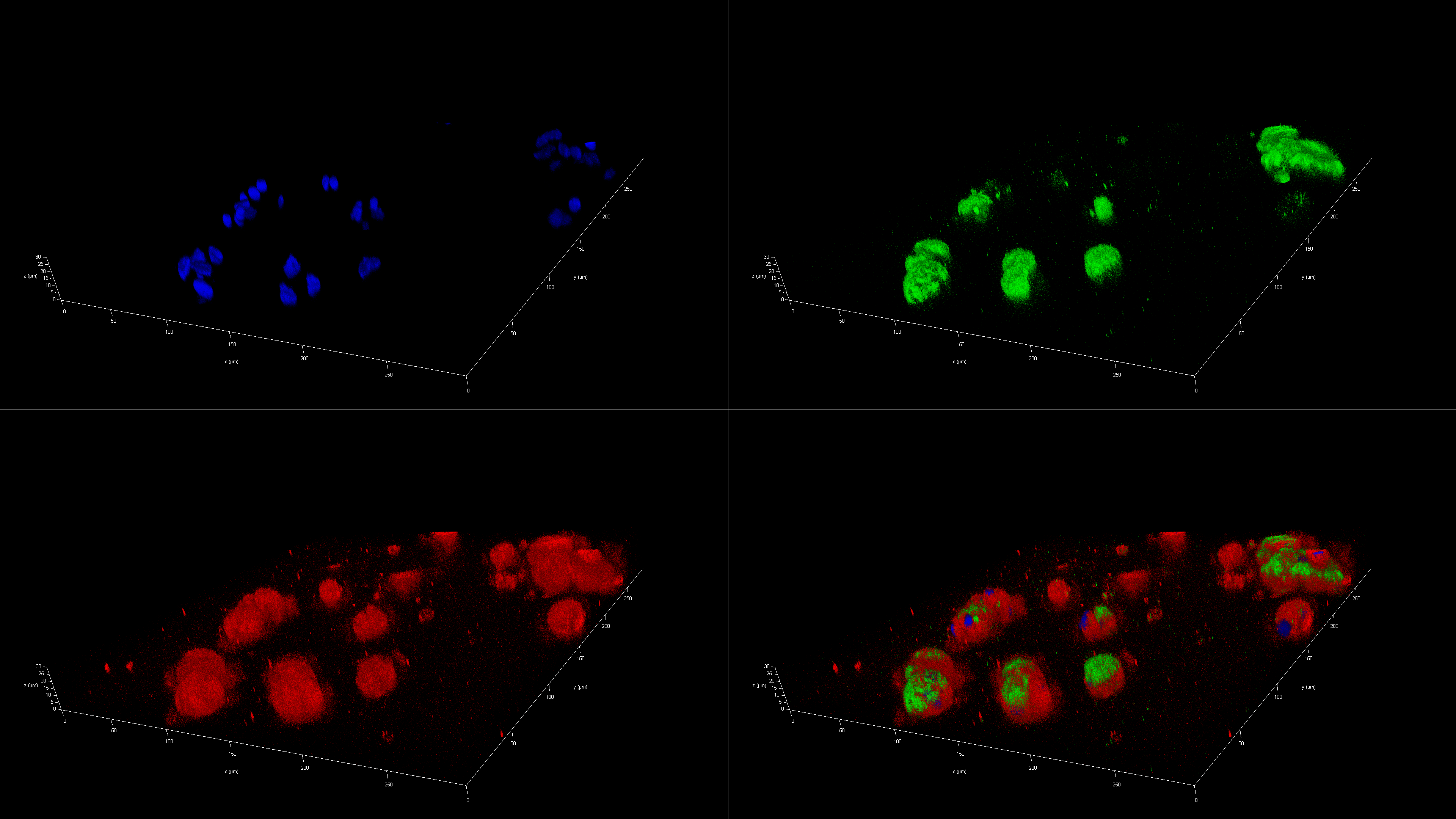

Supplement: Supplementary file 1 [file marinedrugs-23-00268-s001.zip › Figure S10.tif]

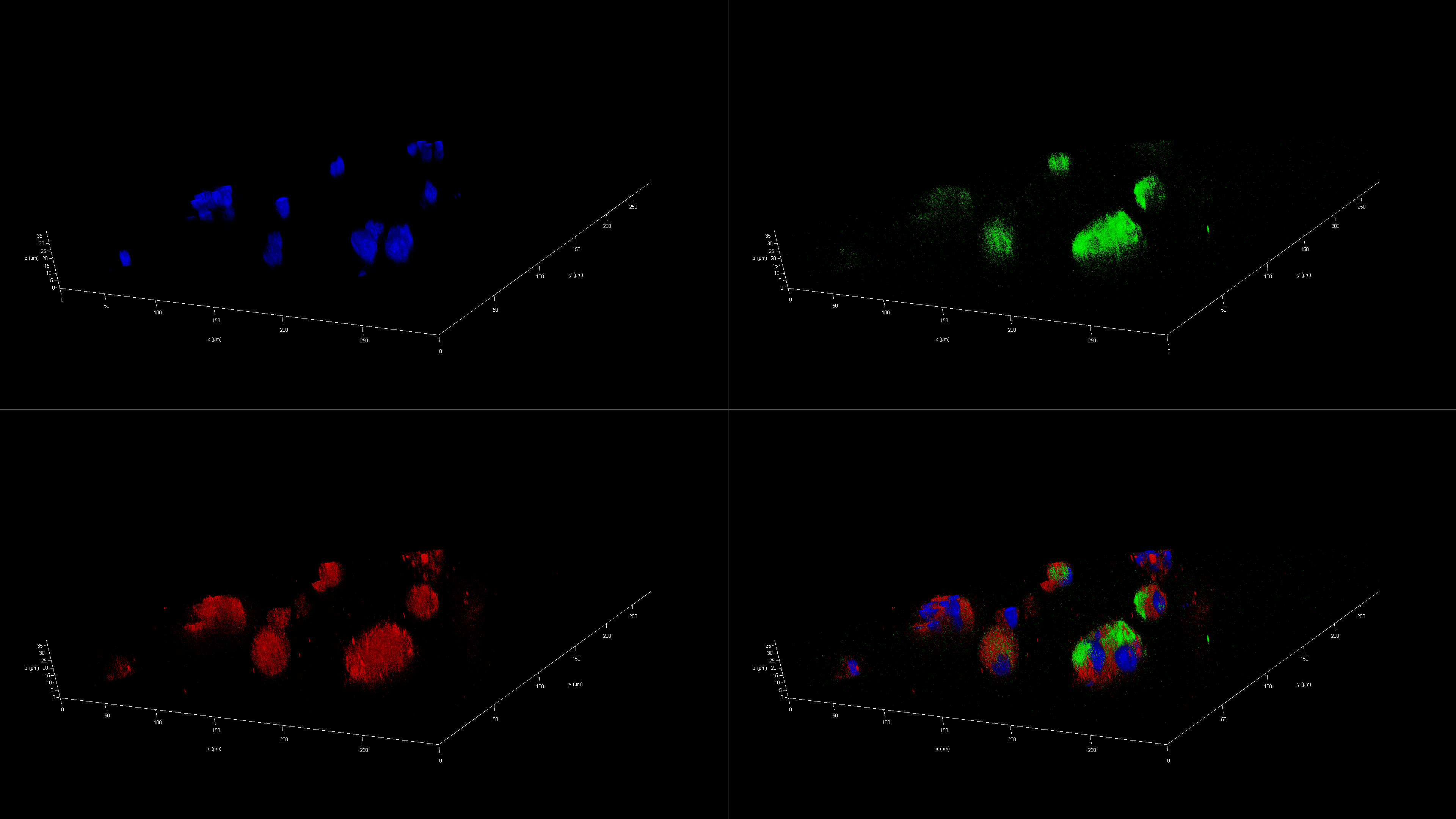

Supplement: Supplementary file 1 [file marinedrugs-23-00268-s001.zip › Figure S11.tif]

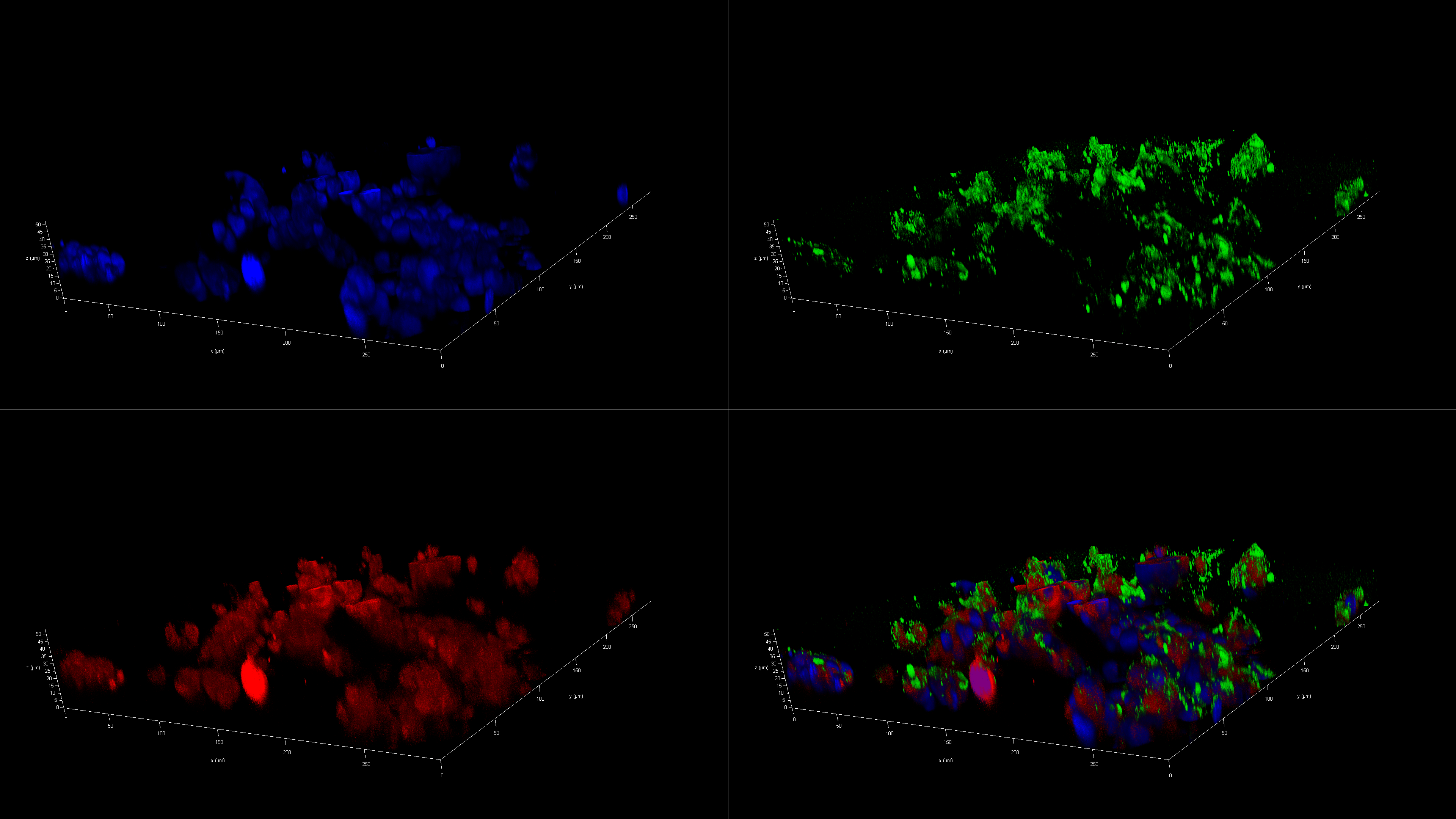

Supplement: Supplementary file 1 [file marinedrugs-23-00268-s001.zip › Figure S12.tif]

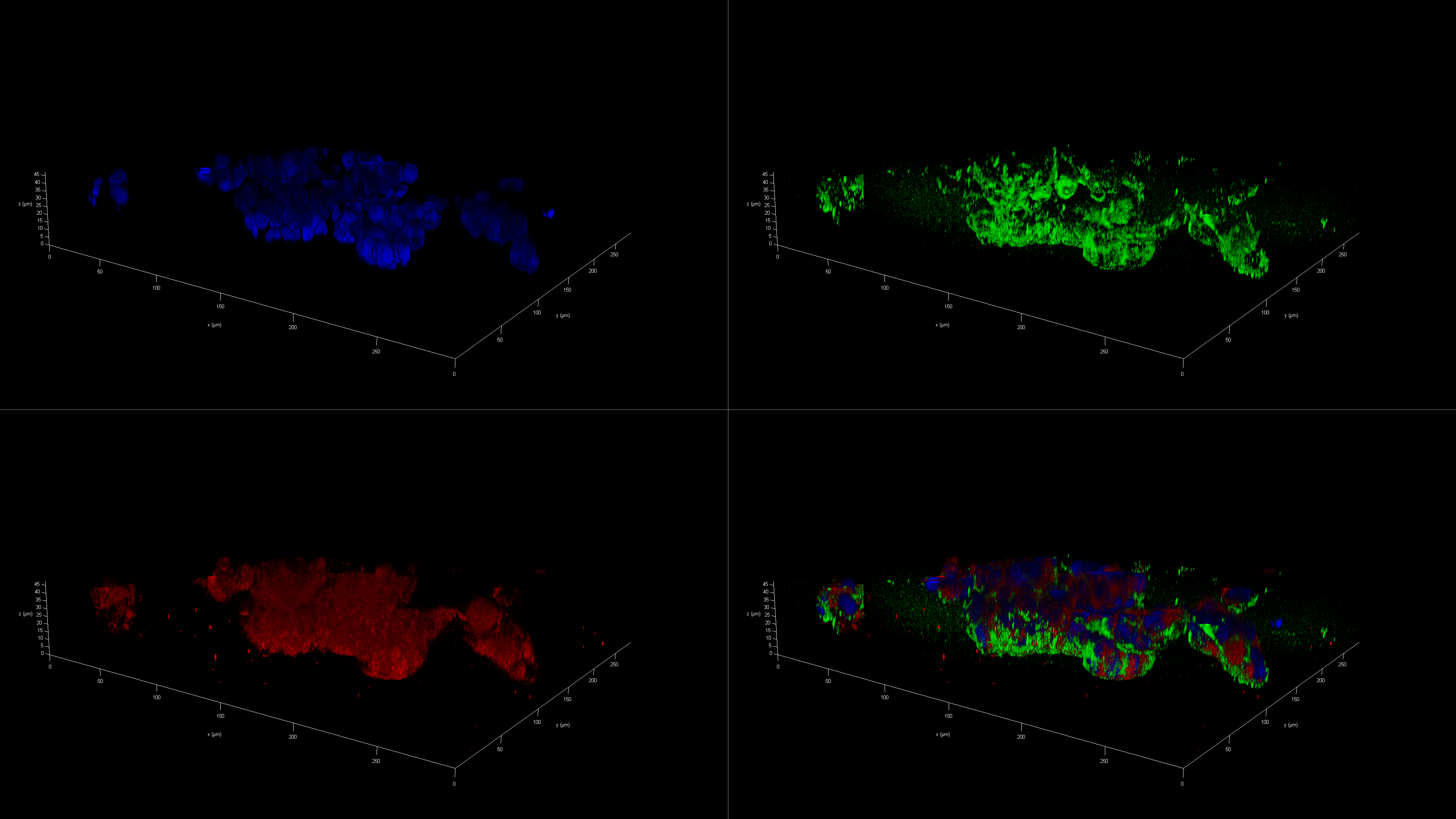

Supplement: Supplementary file 1 [file marinedrugs-23-00268-s001.zip › Figure S13.tif]

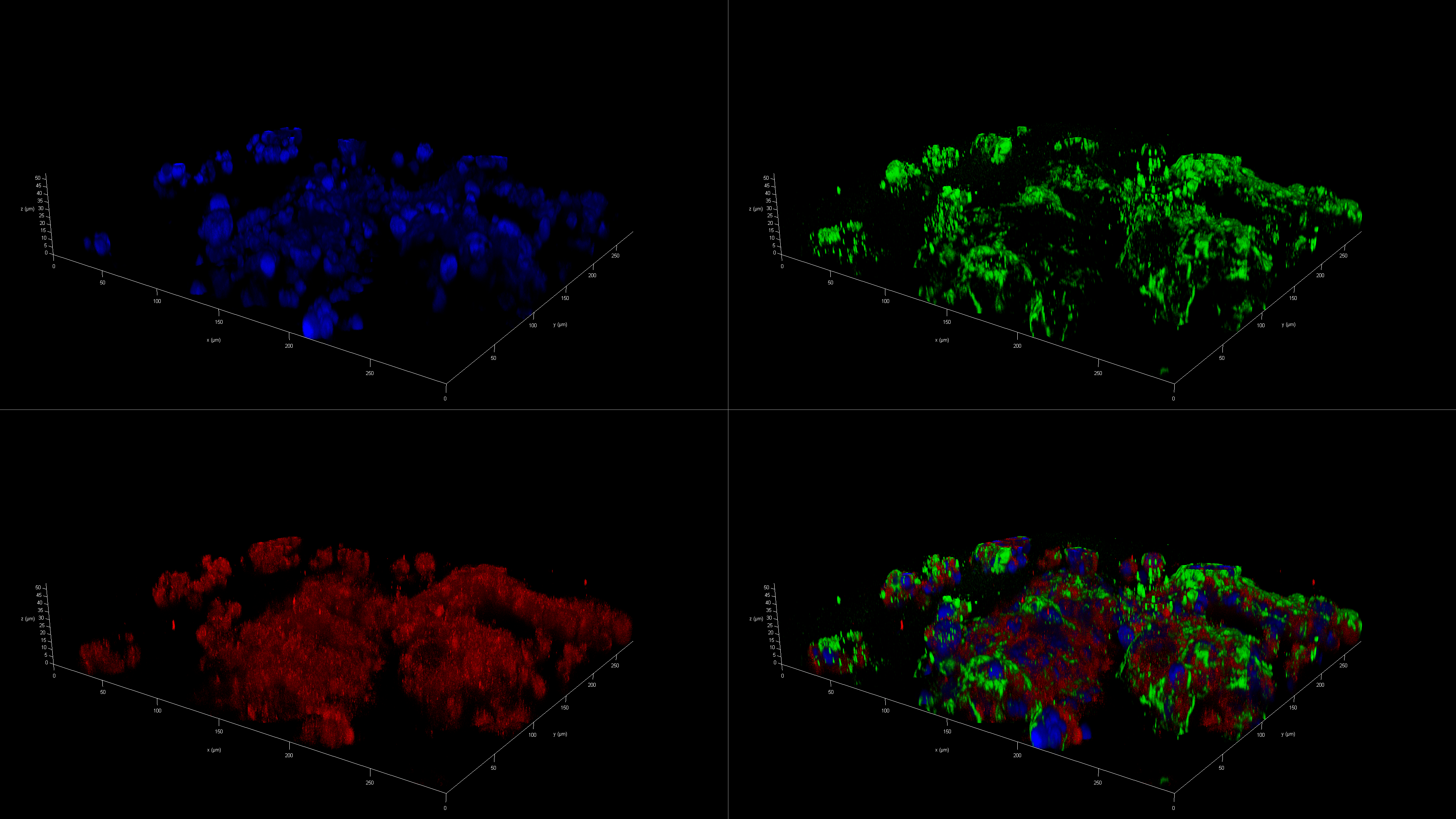

Supplement: Supplementary file 1 [file marinedrugs-23-00268-s001.zip › Figure S14.tif]

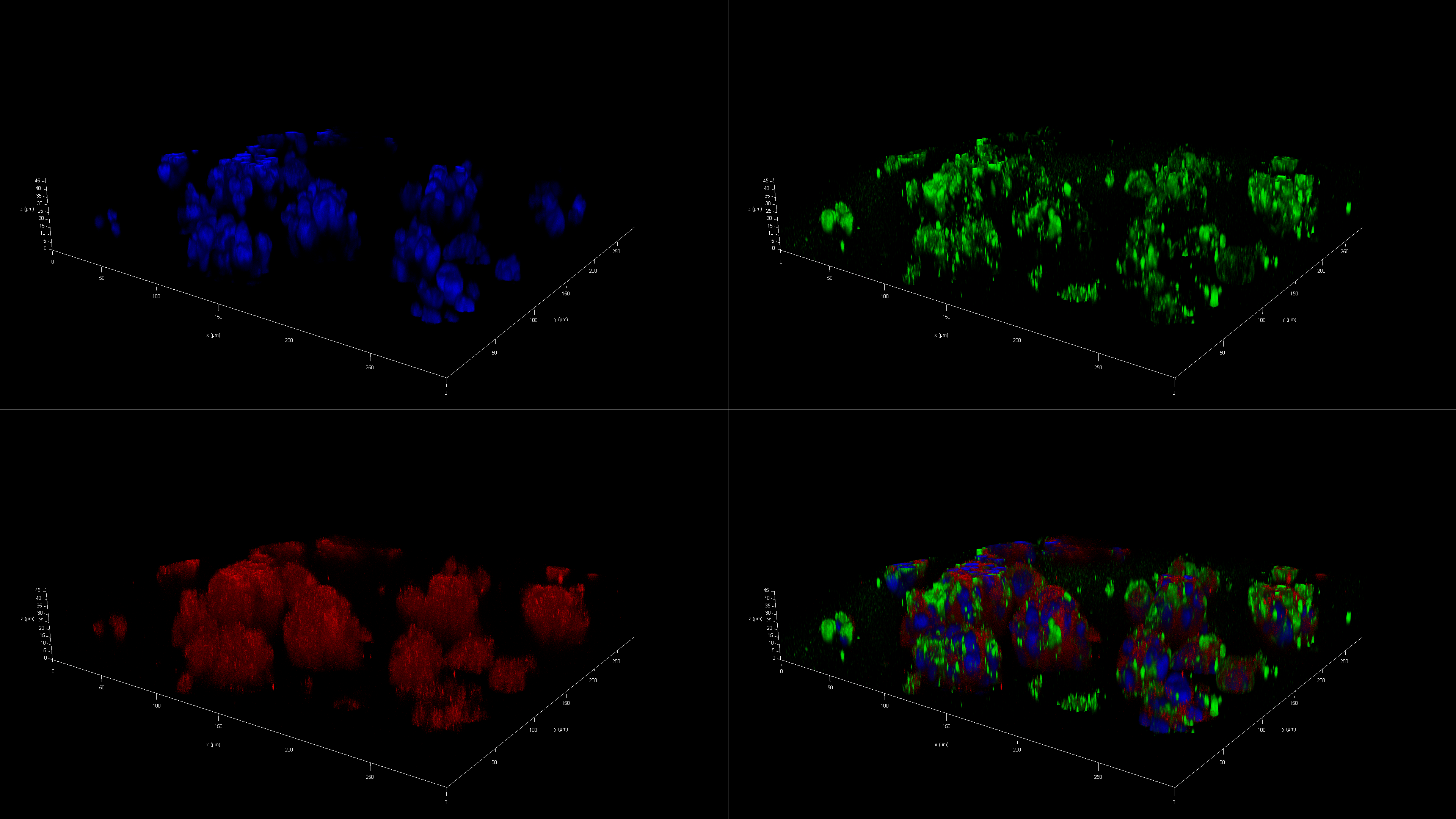

Supplement: Supplementary file 1 [file marinedrugs-23-00268-s001.zip › Figure S15.tif]

**A) EPA**

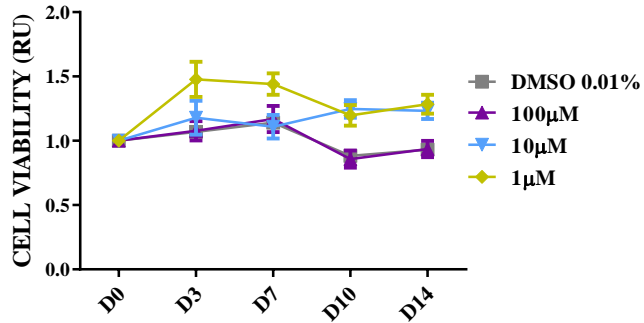

**B) DHA**

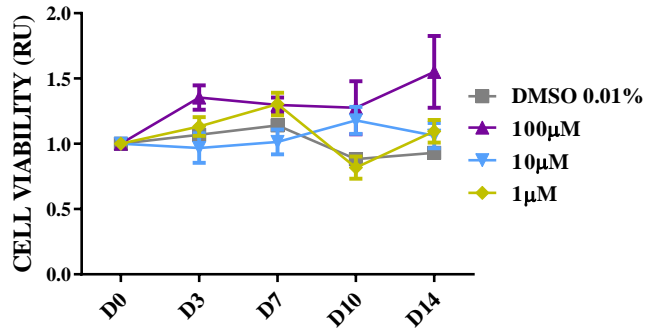

Supplement: Supplementary file 1 [file marinedrugs-23-00268-s001.zip › Figure S16.pdf]

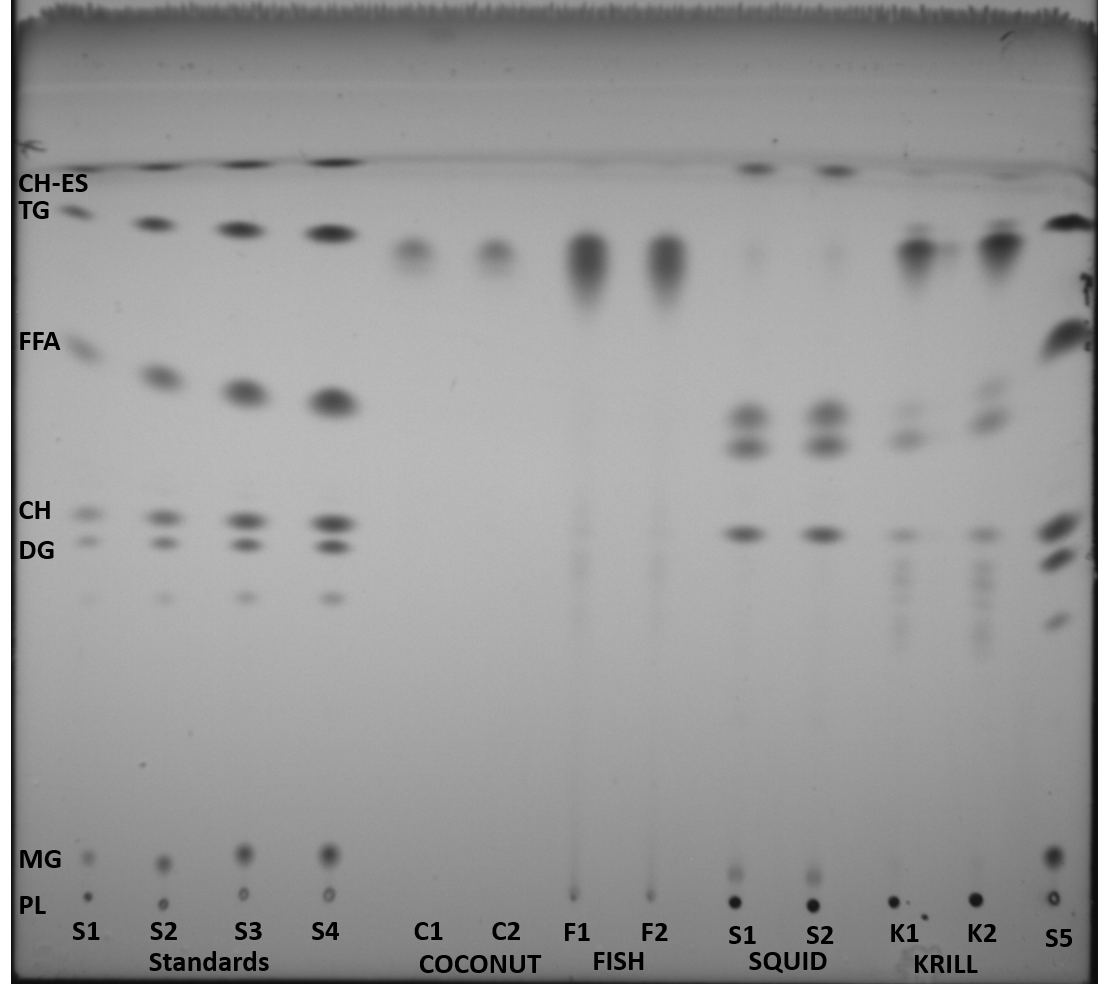

Supplement: Supplementary file 1 [file marinedrugs-23-00268-s001.zip › Figure S17.png]

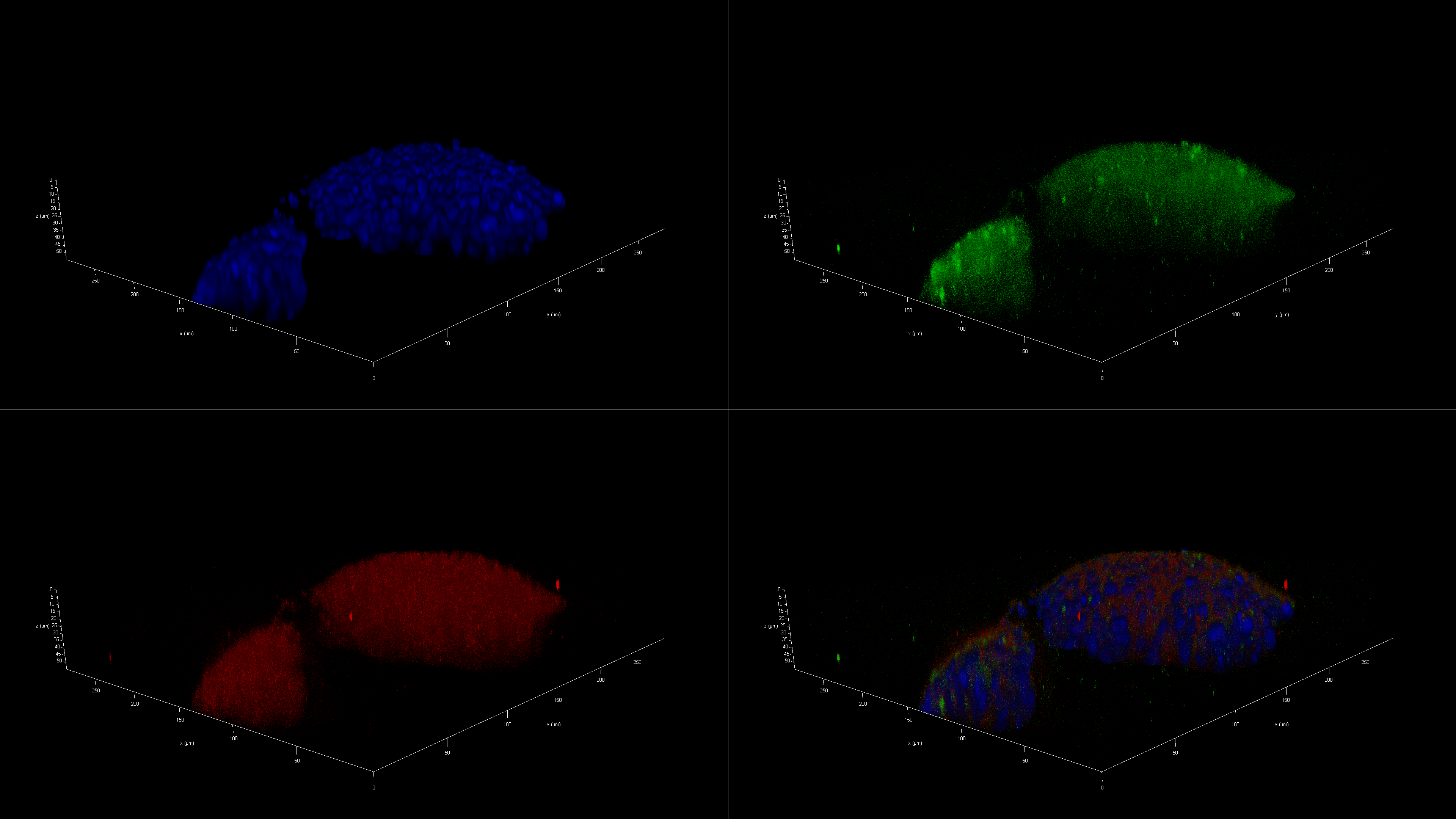

Supplement: Supplementary file 1 [file marinedrugs-23-00268-s001.zip › Figure S2.tif]

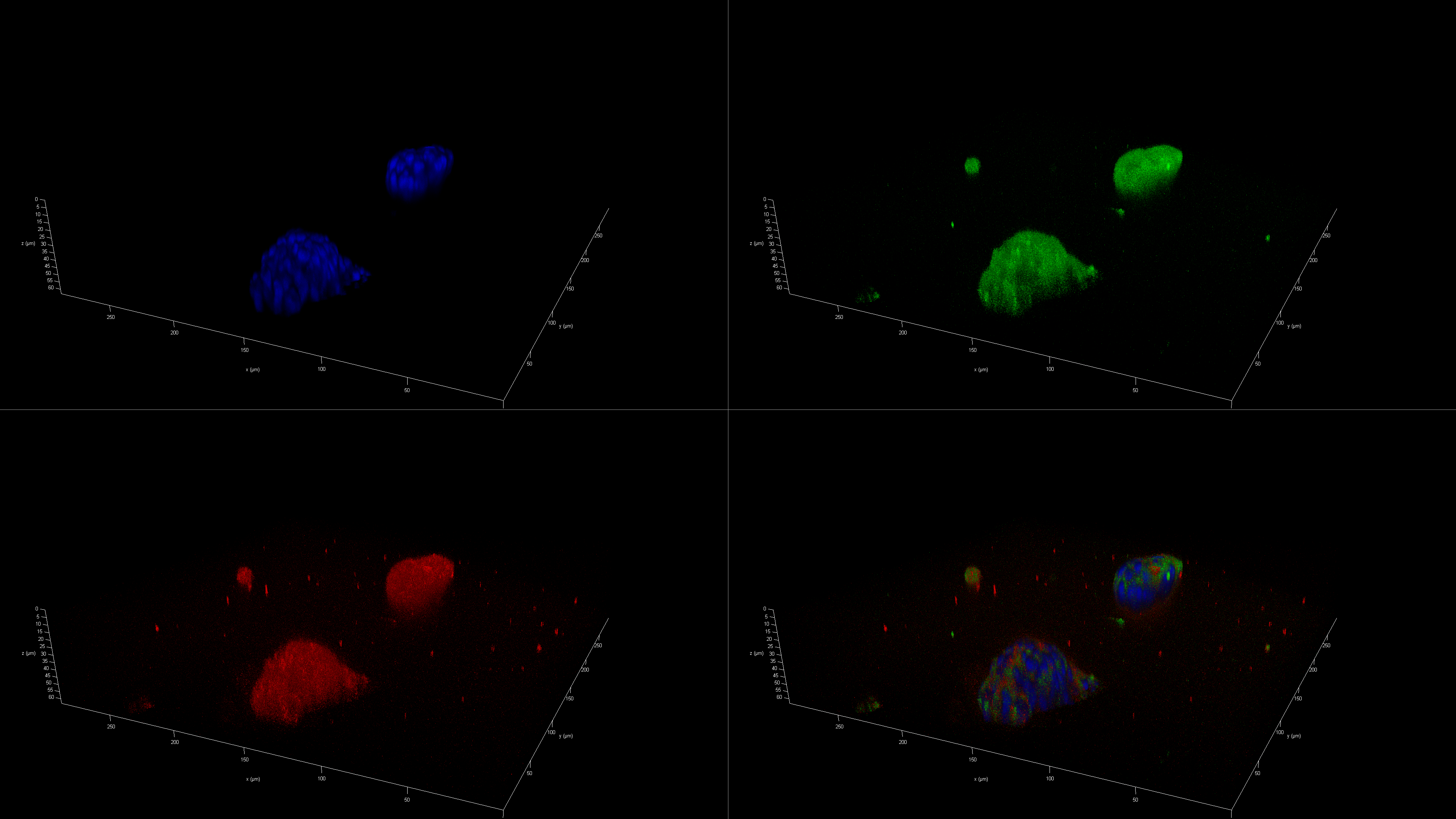

Supplement: Supplementary file 1 [file marinedrugs-23-00268-s001.zip › Figure S3.tif]

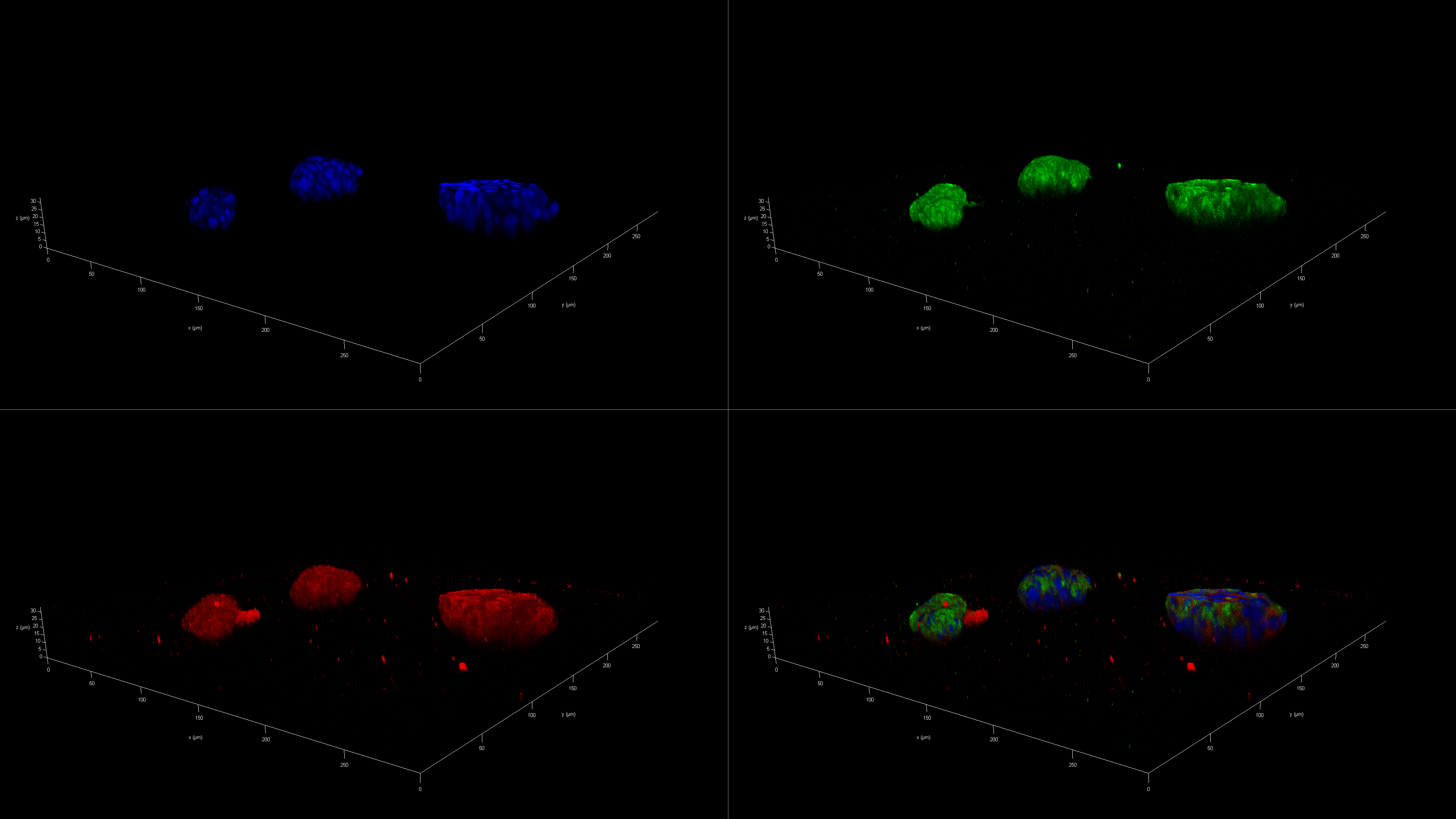

Supplement: Supplementary file 1 [file marinedrugs-23-00268-s001.zip › Figure S4.tif]

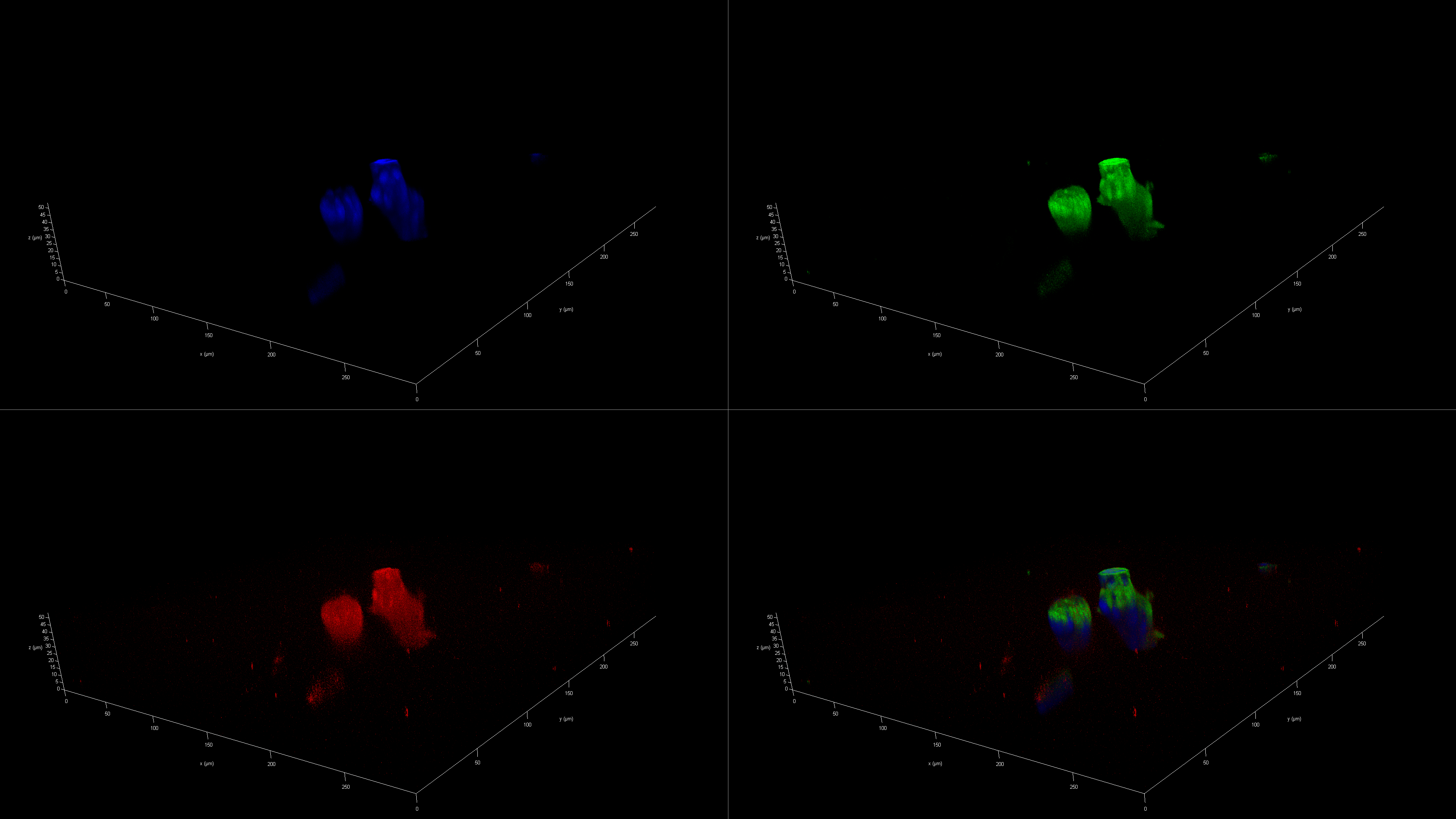

Supplement: Supplementary file 1 [file marinedrugs-23-00268-s001.zip › Figure S5.tif]

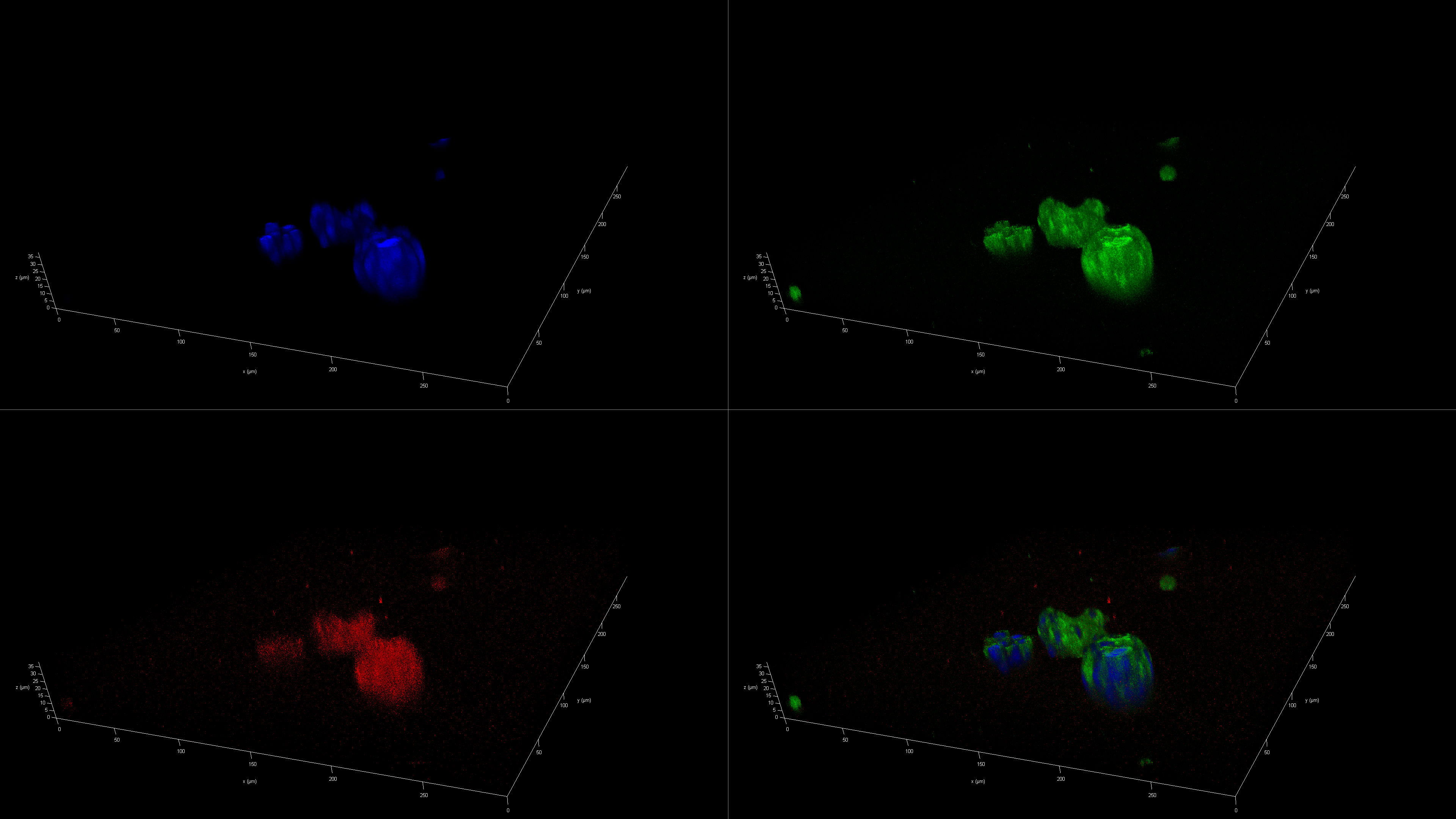

Supplement: Supplementary file 1 [file marinedrugs-23-00268-s001.zip › Figure S6.tif]

**A) EPA**

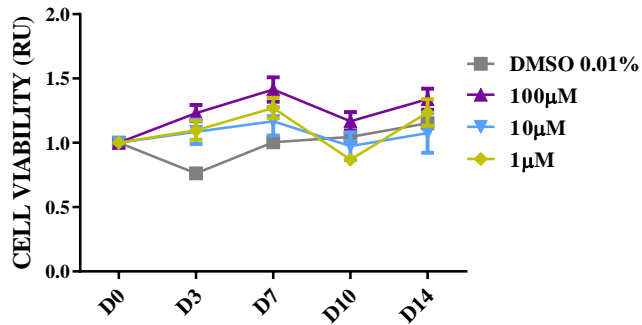

**B) DHA**

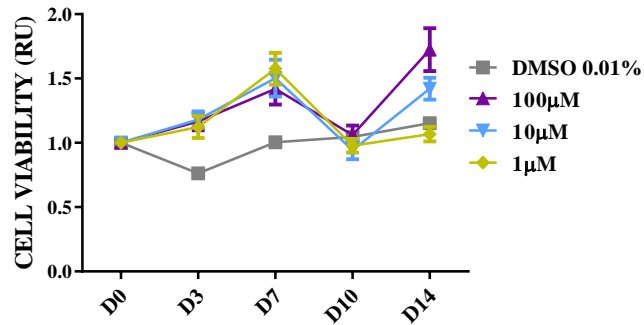

Supplement: Supplementary file 1 [file marinedrugs-23-00268-s001.zip › Figure S7.pdf]

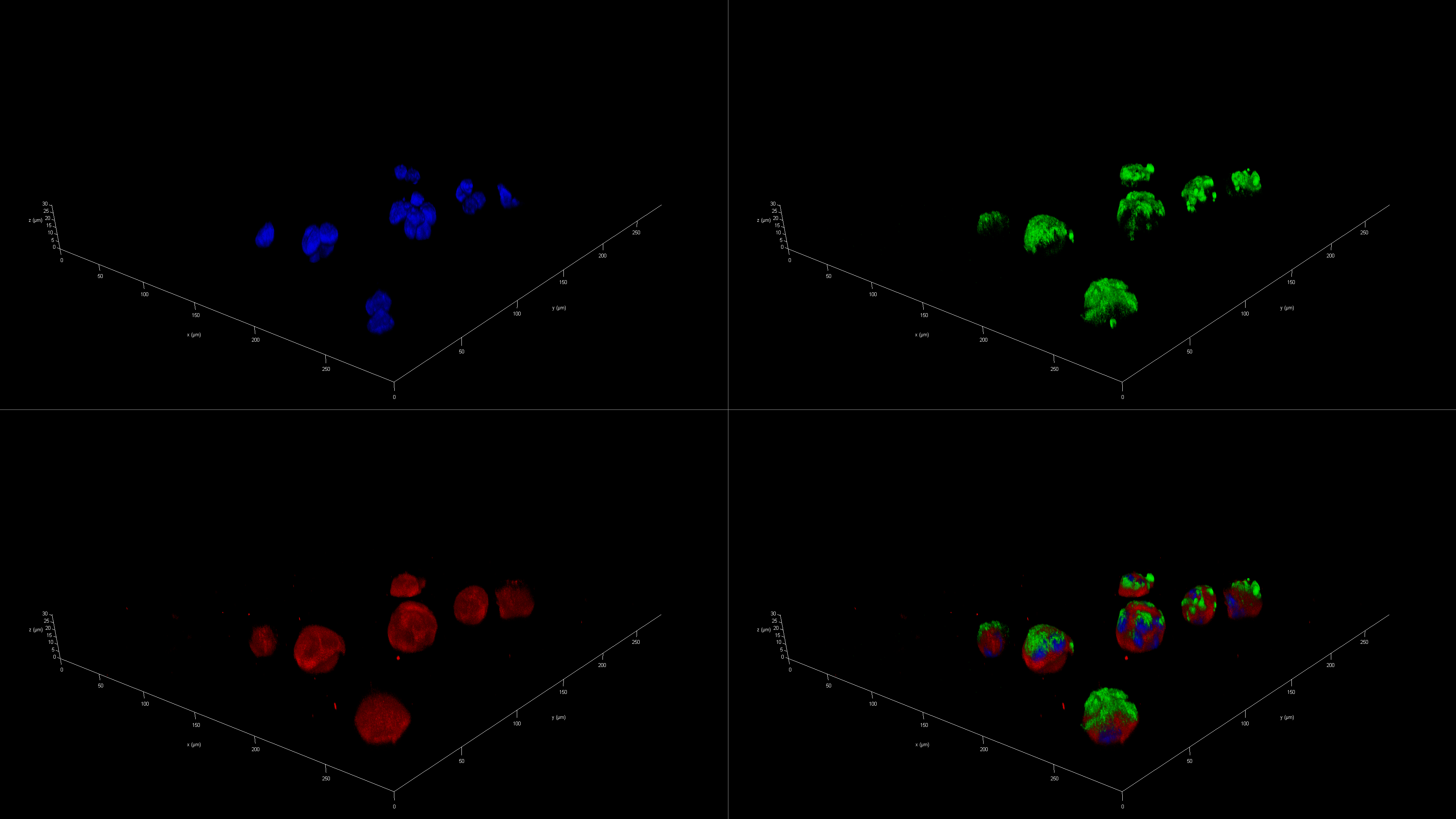

Supplement: Supplementary file 1 [file marinedrugs-23-00268-s001.zip › Figure S8.tif]

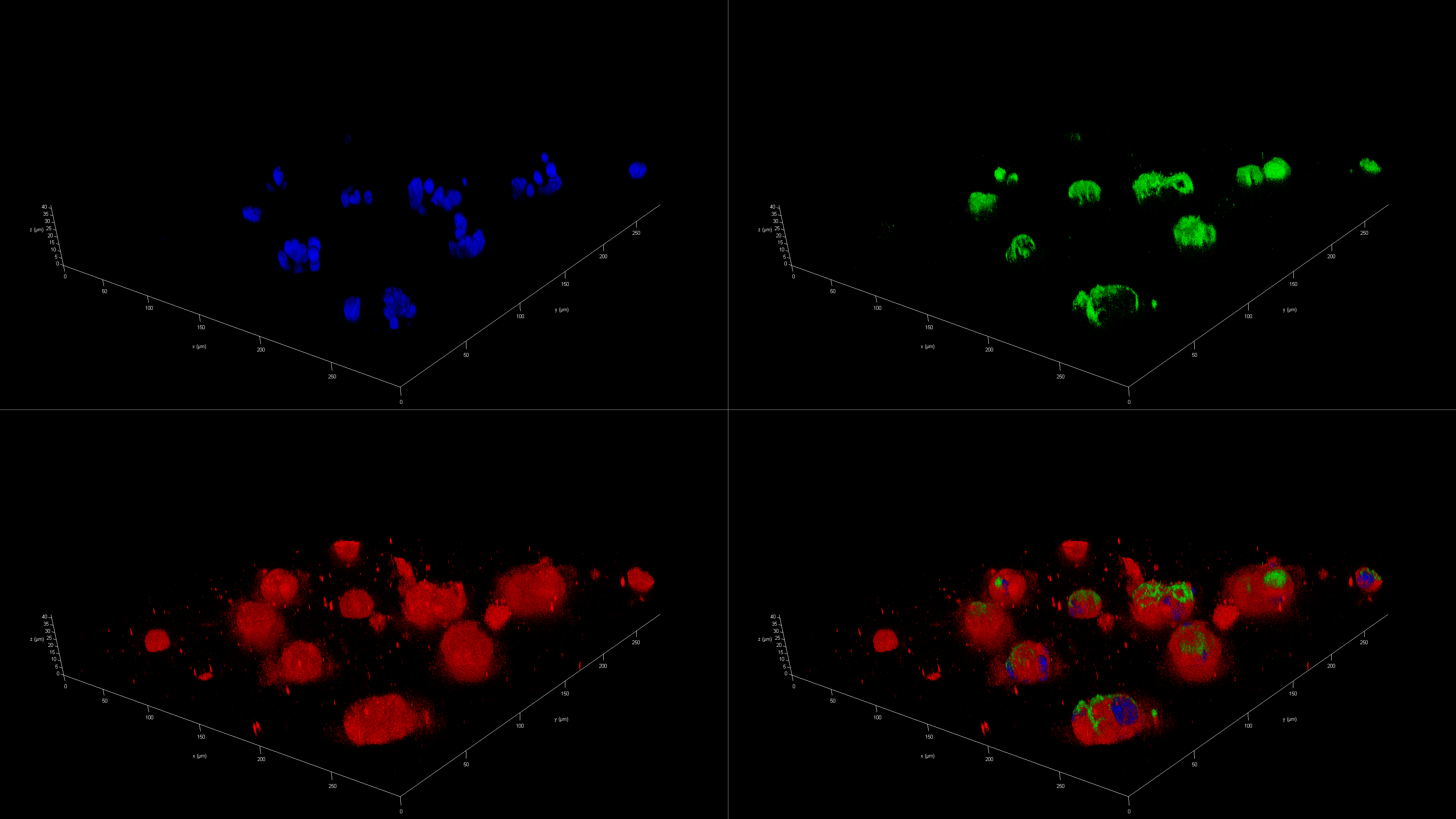

Supplement: Supplementary file 1 [file marinedrugs-23-00268-s001.zip › Figure S9.tif]
